# A systematic evaluation of cell-type-specific differential methylation analysis in bulk tissue

**DOI:** 10.1093/bib/bbaf170

**Published:** 2025-04-16

**Authors:** Shuo Li, Pei Fen Kuan

**Affiliations:** Department of Applied Mathematics and Statistics, Stony Brook University, Nicolls Road, 11794, New York, USA; Department of Applied Mathematics and Statistics, Stony Brook University, Nicolls Road, 11794, New York, USA

**Keywords:** DNA methylation, cell-type-specific differential CpGs, bulk tissue, epigenetics, EWAS

## Abstract

We conducted a systematic assessment of computational models—CellDMC, TCA, HIRE, TOAST, and CeDAR—for detecting cell-type-specific differential methylation CpGs in bulk methylation data profiled using the Illumina DNA Methylation BeadArrays. This assessment was performed through simulations and case studies involving two epigenome-wide association studies (EWAS) on rheumatoid arthritis and major depressive disorder. Our evaluation provided insights into the strengths and limitations of each model. The results revealed that the models varied in performance across different metrics, sample sizes, and computational efficiency. Additionally, we proposed integrating the results from these models using the minimum p-value ($minpv$) and average p-value ($avepv$) approaches. Our findings demonstrated that these aggregation methods significantly improved performance in identifying cell-type-specific differential methylation CpGs.

## Introduction

Epigenomics is a branch of genomics focused on the study of epigenetic modifications, i.e. heritable changes in gene expression that occur without altering the DNA sequence [1]. One of the most commonly studied epigenetic marks is DNA methylation via the epigenome-wide association studies (EWAS) for identifying cytosine-phosphate-guanine (CpG) sites associated with traits like disease status, age, gender, and smoking history. Current leading methods for DNA methylation profiling include the Illumina Infinium HumanMethylation450 (450k) [2] array and the more recent Infinium MethylationEPIC (EPIC) array [3]. The Infinium MethylationEPIC v2.0 Array analyses over 850,000 CpG sites across the human genome, covering key functional regions like gene promoters, enhancers, CpG islands, shores, and open sea regions. It also incorporates updated content reflecting the latest genomic annotations and regulatory elements [4]. Due to the cost-effectiveness and efficiency in capturing average methylation profiles across large cell populations, EWAS are often performed on bulk tissue samples, which are mixtures of different cell types. This creates two key challenges in EWAS. The first challenge is estimating cell type proportions, as variations in cell type composition between samples could be linked to specific phenotypes. Secondly, CpG sites with differential DNA methylation may influence phenotype changes, but these effects may not be present in all cell types, highlighting the need for cell-type-specific differential methylation methods.

In recent years, numerous methods have been developed for detecting cell-type-specific differential expression from bulk tissues, including CellDMC [5], TCA [6], HIRE [7], TOAST [8], and CeDAR [9]. In the next section, we briefly review these methods.

### Cell-type-specific differential methylation algorithms

CellDMC utilizes a linear model that includes interaction terms between the phenotype and pre-estimated cell-type fractions [5]. These interaction terms account for differential methylation specific to individual cell types. The algorithm shows high sensitivity and specificity across a range of simulated and real datasets, surpassing state-of-the-art methods, especially in cases involving bidirectional methylation changes. However, its performance depends heavily on accurate estimation of cell-type fractions and large sample sizes to yield reliable outcomes. Additionally, the requirement for reference methylation profiles across different cell types may limit its application in tissues with poorly characterized cellular compositions. Since CellDMC is based on a linear regression model, the assumption of a Gaussian distribution may be less suitable when sample sizes are small.

TCA [6] introduces an innovative method for identifying cell-type-specific DNA methylation signals from bulk tissue data, bypassing the high costs and technical constraints of cell sorting and single-cell techniques. By using matrix factorization, TCA decomposes bulk methylation data into cell-type-specific components, greatly enhancing the power of epigenetic studies by providing insights without the need for extensive cell-type-specific data collection. This approach has demonstrated substantial improvements in detecting cell-type-specific associations compared to traditional methods. Its robustness is further strengthened by its ability to integrate and refine noisy estimates of cell-type proportions, making it resilient to inaccuracies in initial estimates. However, TCA is computationally intensive, demanding significantly more processing time than CellDMC when applied to the same dataset. This complexity and high computational cost may present challenges for very large datasets or those with numerous cell types. Similar to CellDMC, TCA also assumes a Gaussian distribution, which may be less appropriate for smaller sample sizes.

HIRE [7] employs a hierarchical framework designed to mirror the data generation process in EWAS, facilitating the detection of cell-type-specific associations by modeling the phenotypic effects as multiplicative influences on methylation levels. The algorithm incorporates matrix decomposition and a generalized expectation-maximization approach to estimate cell-type proportions internally. Despite its strengths, HIRE’s performance is sensitive to sample size, with smaller datasets potentially reducing power and precision. Additionally, it is computationally intensive and, like similar methods, assumes a Gaussian distribution, which may be less suitable for smaller sample sizes.

TOAST [8] models bulk data using a linear model framework, where the data is represented as a linear combination of cell-type-specific signals. This approach offers flexibility, enabling the testing of various hypotheses by evaluating linear combinations of the resulting coefficients, such as comparing gene expression between cell types or conditions within a specific cell type. While TOAST is computationally efficient compared to permutation-based methods, the challenge of accurately modeling multiple cell types and handling large datasets remains significant. Moreover, its performance may be affected by high inter-individual heterogeneity.

CeDAR [9] utilizes a hierarchical Bayesian model for the detection of cell-type-specific signals. Its strength lies in leveraging the cell type hierarchy to improve the detection of differential signals. By utilizing correlations among related cell types, CeDAR increases detection power, particularly for low-abundance cell types.

Several studies have compared these algorithms using bulk gene expression data [10–12]. In this paper, we provide a comprehensive comparison of these methods specifically for cell-type-specific differential methylation analysis via simulations and a case study. In addition to focusing on DNA methylation data, a key distinction of our work is that we assess whether aggregating the results from these methods yields improvements over using individual methods.

## Methods

### Reference datasets

We utilized the following lists of DNA methylation data sets generated on purified cells pools from the Gene Expression Omnibus (GEO, https://www.ncbi.nlm.nih.gov/geo) and the EMBL’s European Bioinformatics Institute (https://www.ebi.ac.uk) listed below to generate in-silico mixtures:

- Illumina 450k data of 6 epithelial and 10 fibroblast cell lines [13] (GSE31848)
- Illumina 450k data of 28 monocytes, 71 CD4T cells, and 8 B-cells [14] (GSE59250)
- Illumina 450k data of 6 CD4T cells, 4 B-cells, and 5 monocytes [15] (GSE71244)
- Illumina 450k data of 8 CD4T cells [16] (GSE50222)
- Illumina 450k data of 214 CD4T cells and 1,202 monocytes [17] (GSE56047)
- Illumina 450k data of 6 B-cells, 6 CD4T cells, and 6 monocytes [18] (E-MTAB-2145)

The methylation datasets were normalized using the Beta-Mixture Quantile (BMIQ) method [19]. To ensure numerical stability, we applied a threshold of $\epsilon = 10^{-6}$, replacing all occurrences of zeros and ones in the methylation data with $\epsilon $ and $1-\epsilon $, respectively. A detection p-value threshold of $10^{-16}$ was used, and probes with p-values exceeding this threshold were set as missing. We removed samples with a call rate below 95% and probes with a call rate below 90%. After filtering, missing methylation data were imputed using the nearest neighbor averaging method via the impute.knn function from the impute R package [20, 21].

We examined the Pearson correlations between samples within each dataset and cell type, identifying several with significantly lower correlations. Following this, we conducted a cell purity check using the HEpiDISH tool [22]. Samples with a cell type fraction exceeding 95% were classified as pure, while those below this threshold were filtered out. Notably, none of these samples were used in constructing the DNA methylation references for HEpiDISH, ensuring an unbiased estimation of cell type proportions. The final dataset used to generate in-silico mixtures consisted of 3 epithelial, 6 fibroblast, 231 CD4T cell, 945 monocyte, and 12 B-cell samples, totaling 1,197 samples across 484,468 CpG sites.

### Data generation

We simulated data for 10,000 CpGs and used HEpiDISH [22] to estimate cell type proportions. Of these, 904 CpGs were included in the reference libraries used by both EpiDISH and HEpiDISH, while the remaining 9,096 CpGs were randomly selected from the existing set of 484,468 CpGs for each replication.

To assess model performance across different cell type proportions, we generated cell type proportions $f_{k}$ for each cell type $k$ using the following distributions: Epithelial $\sim U(0.3, 0.4)$, Fibroblasts $\sim U(0.35, 0.45)$, CD4T $\sim U(0.1, 0.2)$, Monocytes $\sim U(0, 0.1) $, B-cells $ \sim U(0, 0.1)$

Next, we calculated the mean ($\mu _{ck}$) and variance ($\sigma ^{2}_{ck}$) for each cell type $k$ and CpG $c$ using the 1,197 pure cell sample datasets. Due to the limited number of pure epithelial and fibroblast samples, we performed Levene’s test on each CpG to determine whether using a common variance across all cell types was appropriate. The test results indicated that a common variance was reasonable. Therefore, for each CpG, we computed a pooled variance ($\sigma ^{2}_{c}$) across pure CD4T, monocyte, and B-cell samples. The effect size ($s$) was determined using the formula $s_{c}=\text{SNR} \cdot \sigma _{c}$, where SNR is the signal-to-noise ratio. We considered SNR values ranging from 7 to 15, in increments of 2, and two sample size settings ($n=100$ and $n=200$). For each sample size, we simulated binary phenotypes with a 1:1 case-control ratio. Each SNR$\times n$ setting was replicated 50 times for robustness.

For CpGs exhibiting cell-type-specific effects (i.e. a mean difference between case and control), the cell-type-specific methylation profiles $\vec{X}_{ck}$ for the cases were simulated as:


\begin{align*} & \vec{X}_{ck} \sim N\left( \mu_{c k} + s_{c}, \sigma_{c}^{2} \right) \end{align*}

We set non-overlapping subsets of 500 CpGs to exhibit cell-type-specific effects in epithelial, CD4T, and monocyte cells, respectively, while fibroblasts and B-cells were assigned no cell-type-specific effects. The cell-type-specific methylation profiles for the controls were simulated as:


\begin{align*} & \vec{X}_{ck} \sim N\left( \mu_{c k}, \sigma_{c}^{2} \right) \end{align*}

Finally, the cell specific profiles $\vec{X}_{ck}$ were multiplied by the simulated cell type fraction $f_{k}$ to generate in-silico mixtures:


\begin{align*} & \vec{X}_{c} = \sum_{k=1}^{K} \vec{X}_{c k} f_{k} \end{align*}

### Model comparisons

We compared five methods for detecting cell-type-specific differential methylation: CellDMC [5], TCA [6], HIRE [7], TOAST [8], and CeDAR [9]. Cell-type proportions were estimated using HEpiDISH [22]. As the goal was to evaluate the performance of these methods in identifying cell-type-specific differences, each method was provided with the true cell proportions.

Additionally, ensemble learning—where predictions from multiple algorithms are combined—has been shown to improve accuracy in machine learning [23]. Drawing from this idea, we considered two approaches for aggregating results from the different cell-type-specific differential methylation methods. The first approach is the “p-values averaging” (avepv) method. Denoting the p-values for testing differential methylation in cell type $k$ and CpG site $c$ obtained from CellDMC, TCA, HIRE, TOAST, and CeDAR as $p_{ck1}, p_{ck2}, p_{ck3}, p_{ck4}$, and $p_{ck5}$, respectively, avepv is calculated as:


\begin{align*} &\text{avepv}_{ck}=\Phi\left(\frac{1}{5}\sum_{i=1}^{5}\Phi^{-1}(p_{cki})\right)\end{align*}

where $\Phi ()$ is the cumulative distribution function (CDF) of a standard normal distribution.

The second approach, “minimum p-value” (minpv), is defined as:


\begin{align*} &\text{minpv}_{ck}=F\left(\min_{i}(p_{cki})\right)\end{align*}

where $F()$ is the CDF of a Beta distribution with parameters 1 and 5.

All methods, except CeDAR, output estimated p-values. CeDAR instead identifies cell-type-specific differentially methylated CpG sites using posterior probabilities. The authors suggested classifying CpGs with a posterior probability greater than 0.95 as differentially methylated. To ensure comparability with the other methods, which are evaluated using a false discovery rate (FDR) threshold, we applied a Bayesian FDR approach, as outlined by Newton *et al.* [24]. In the $avepv$ and $minpv$ approaches, we set $p_{ck5}=1-$posterior probability.

In our simulation studies, we applied an FDR threshold of 0.05 and evaluated the methods using several performance metrics: empirical FDR, sensitivity, specificity, area under the receiver operating characteristic curve (AUROC), F1 score, false omission rate (FOR), and the estimated type I error rate. Additionally, we reported the running time for each method to assess computational efficiency. Runtime was analysed across different combinations of the number of CpGs ($p = 10k, 50k, 100k, 200k, 300k,$ and $450k$) and sample sizes ($n = 100, 200, 500,$ and $700$).

### Benchmarking on Illumina MethylationEPIC data

We extended our simulation studies to purified cell pools from FlowSorted.Blood.EPIC (GSE110554), which includes methylation data profiled on Illumina MethylationEPIC arrays for 6 neutrophils, 6 monocytes, 6 B-cells, 7 CD4T cells, 6 CD8T cells, and 6 natural killer (NK) cells. The dataset was preprocessed using a pipeline similar to that applied to 450K datasets. For each replication, we simulated data for 20,000 CpGs selected from the existing set of 863,827 CpGs. Of these, 315 CpGs were included in the reference library centDHSbloodDMC.m from EpiDISH, while the remaining 19,685 CpGs were randomly selected from the existing set of 863,512 CpGs for each replication.

We generated cell proportions $f_{k}$ for each cell type using the following distributions:

Neutrophils$\sim U(0.4, 0.6)$, CD4T$\sim U(0.2, 0.3)$,

CD8T$\sim U(0.05, 0.15)$, Monocytes$\sim U(0, 0.1)$,

B-cells$\sim U(0, 0.1)$, NK$\sim U(0, 0.1)$

We considered four settings:

- Setting 1a: Non-overlapping subsets of 1,000 CpGs were assigned cell-type-specific effects in neutrophils, CD4T, CD8T, and monocytes, while B-cells and NK cells were not assigned any cell-type-specific effects. This resulted in a total of 4,000 CpGs with cell-type-specific effects.
- Setting 1b: Among these, 500 CpGs with cell-type-specific effects were shared between CD4T and CD8T, reducing the total number of unique CpGs with cell-type-specific effects to 3,500.
- Setting 2a: Cell-type-specific effects were assigned to 1,000 CpGs in CD4T, CD8T, and monocytes, while neutrophils, B-cells, and NK cells had none, resulting in a total of 3,000 CpGs with cell-type-specific effects.
- Setting 2b: Similar to Setting 2a, but with 500 CpGs shared between CD4T and CD8T, reducing the total to 2,500 CpGs with cell-type-specific effects.

## Results

**Figure 1:**
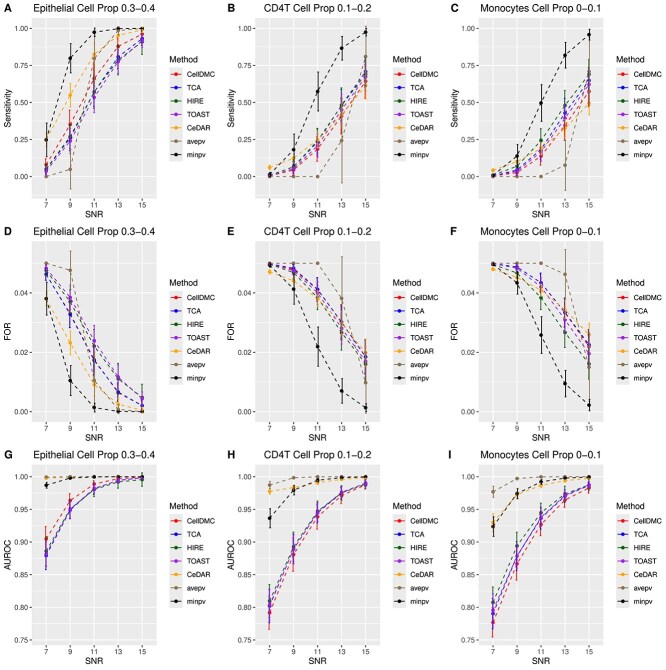
Sensitivity, FOR, and AUROC across different methods and cell type proportions for $n=100$ based on Illumina 450k benchmarking datasets. A–C. Sensitivity plots for cell-type-specific differential methylation analysis in epithelial, CD4T, and monocytes, respectively. D–F. FOR plots. G–I. AUROC plots.

**Figure 2:**
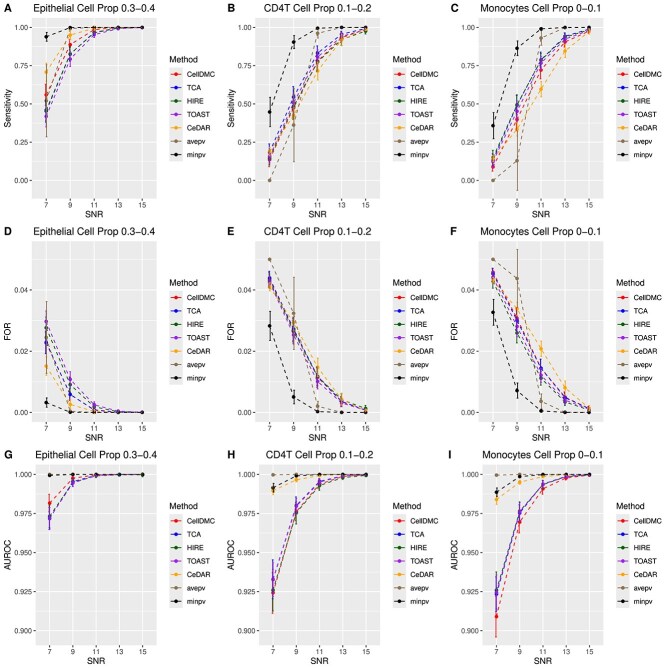
Sensitivity, FOR, and AUROC across different methods and cell type proportions for $n=200$ based on Illumina 450k benchmarking datasets. A–C. Sensitivity plots for cell-type-specific differential methylation analysis in epithelial, CD4T, and monocytes, respectively. D–F. FOR plots. G–I. AUROC plots.

We first present the results from simulation studies using Illumina 450k benchmarking datasets. Figures 1 and 2 display the sensitivity, false omission rate (FOR), and area under the receiver operating characteristic curve (AUROC) for the five individual methods, as well as the two proposed aggregation methods, at sample sizes of $n=100$ and $n=200$, respectively. Specificity and F1 score are provided in Supplementary Figs 1 and 2. In general, as cell-type proportions and the signal-to-noise ratio (SNR) increase, sensitivity, AUROC, and F1 score improve, while FOR decreases. Specificity remains consistently high ($>0.99$) for all methods. Additionally, a larger sample size enhances performance, as reflected in higher sensitivity, AUROC, and F1 score, and a lower FOR for $n=200$ compared to $n=100$.

All individual methods achieve high AUROC values ($>0.9$), with CeDAR showing the highest AUROC across all cell types and SNR levels. CeDAR also excels in sensitivity, F1 score, and FOR in epithelial cells (where cell-type proportions range from 0.3 to 0.4) at lower SNR levels, with CellDMC ranking second. However, CeDAR’s performance decreases in CD4T cells and monocytes (where cell-type proportions range from 0 to 0.2), especially in terms of sensitivity, F1 score, and FOR for $n=200$ (Supplementary Fig. 2), while CellDMC maintains more consistent performance across these cell types. The performance metrics for TCA, HIRE, and TOAST—sensitivity, specificity, F1 score, FOR, and AUROC—are generally comparable and stable.

**Figure 3:**
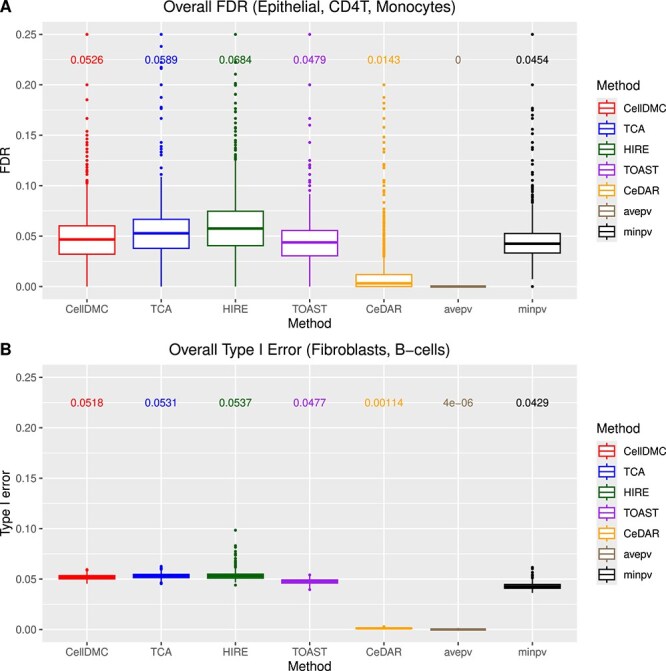
A. Boxplots of empirical FDR across different methods aggregating results for for cell-type-specific differential methylation analysis in epithelial, CD4T, and monocytes, and SNRs for $n=100$ based on Illumina 450k benchmarking datasets. B. Boxplots of empirical type I error across different methods aggregating results for cell-type-specific differential methylation analysis in fibroblasts and B-cells, and SNRs for $n=100$ based on Illumina 450k benchmarking datasets. The printed numbers are the mean values of each method.

Figure 3A and Supplementary Fig. 3A present the empirical false discovery rate (FDR) for $n=100$ and $n=200$, respectively, aggregated across cell types with true differential methylation (i.e. epithelial cells, CD4T cells, and monocytes) and varying SNR levels. Most methods effectively control the FDR, with the exception of HIRE and TCA, which display inflated empirical FDR values at $n=100$ (mean = 0.0684 and 0.0589, respectively). HIRE also exhibits elevated FDR at $n=200$ (mean = 0.0612). For cell types without differential methylation (i.e. fibroblasts and B-cells), Fig. 3B confirms that all methods effectively control the Type I error rate.

The aggregation approaches, particularly the $minpv$ method, stand out for their consistently strong performance across all scenarios. The $minpv$ approach delivers the highest overall performance, while the $avepv$ method tends to show lower sensitivity, though this is balanced by its lower empirical FDR and its achievement of the highest AUROC.

**Figure 4:**
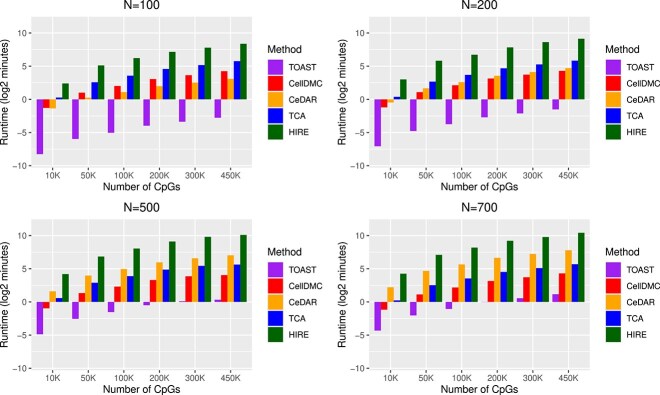
Average elapsed time in log2 minutes for different methods for simulation study $n=100, 200, 500, 700$ and number of CpGs$=10K, 50K, 100K, 200K, 450K$.

Figure 4 illustrates the runtime of each model across varying combinations of CpG counts and sample sizes, measured on an Intel(R) Xeon(R) CPU E5-2620 v2 @ 2.10GHz. Among the methods, TOAST consistently demonstrates the fastest runtime across all scenarios, with CellDMC performing well for sample sizes $n \geq 200$. CeDAR is efficient for $n = 100$ and $200$, but its runtime increases substantially with larger sample sizes. Both CellDMC and TCA exhibit stable runtimes across different sample sizes, scaling linearly with the number of CpGs. In contrast, HIRE is significantly slower than the other methods, requiring considerably longer runtimes and substantial computational memory due to the generalized EM algorithm utilized in its implementation.

Based on the results, we also include $avepv$ and $minpv$ that aggregate results from different subsets of methods. Specifically,

- avepv_M12 and minpv_M12: These aggregate results from CellDMC and TCA, as these methods are specifically designed for methylation data and demonstrate stable performance.
- avepv_M124 and minpv_M124: These aggregate CellDMC, TCA, and TOAST, with TOAST included for its fast runtime.
- avepv_M125 and minpv_M125: These aggregate CellDMC, TCA, and CeDAR, with CeDAR included due to its strong individual performance in terms of AUROC.

**Figure 5:**
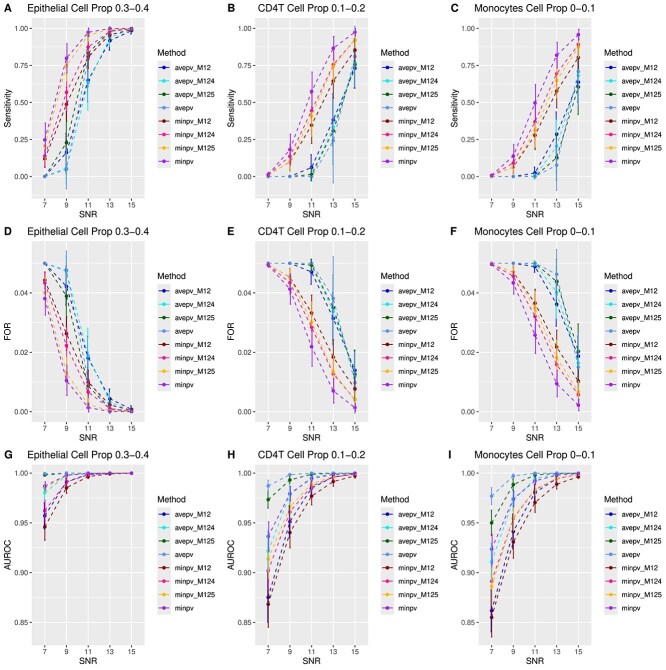
Sensitivity, FOR, and AUROC across different result aggregation methods and cell type proportions for $n=100$ based on Illumina 450k benchmarking datasets ($minpv$ and $avepv$ refer to aggregating results from all five methods). A–C. Sensitivity plots for cell-type-specific differential methylation analysis in epithelial, CD4T, and monocytes, respectively. D–F. FOR plots. G–I. AUROC plots.

Figure 5 and Supplementary Fig. 4 display the sensitivity, FOR, and AUROC for these additional $avepv$ and $minpv$ approaches. Overall, the $minpv$ methods exhibit higher sensitivity and lower FOR compared to the $avepv$ methods. However, the $avepv$ methods tend to achieve higher AUROC. Notably, among the $minpv$ approaches, the version that aggregates all five methods shows the best overall performance. Supplementary Figs 5 and 6 zoom in on the results for CellDMC, TCA, and CeDAR, illustrating that the $minpv$ approach, which combines these three methods, provides a significant improvement over each individual method.

Results from simulation studies using Illumina Methylation-EPIC benchmarking datasets are presented in Supplementary Figs 7–30, largely aligning with findings from simulation studies based on Illumina 450K benchmarking datasets. Notably, we observed greater inflation in the false discovery rate (FDR) for HIRE, leading to inflated FDR in the $minpv$ approach, which aggregates all methods (Supplementary Figs 15 and 17). However, when HIRE was excluded from $minpv$, FDR was effectively controlled (Supplementary Figs 23 and 25).

In Setting 1b, where 500 CpGs with cell-type-specific effects were shared between CD4T and CD8T, and neutrophils were the dominant cell type with cell-type-specific effects (i.e. had the largest relative proportion), we observed improved sensitivity of CeDAR in detecting cell-type-specific CpGs in CD4T (for $n=100$ and $n=200$) and in CD8T (for $n=200$) (Supplementary Figs 7 and 9). However, this improvement was not observed in Setting 2b, where CD4T was the dominant cell type with cell-type-specific effects (Supplementary Figs 8 and 10). On the other hand, HIRE showed substantial improvement in detecting cell-type-specific CpGs in both CD4T and CD8T in Settings 1b and 2b, compared to Settings 1a and 2a, respectively. Although one of CeDAR’s advantages is its ability to account for the cell type hierarchy to enhance detection of cell-type-specific CpGs by borrowing information across cell types, our simulation studies did not show a substantial improvement using this approach.

## Case studies

### Case study 1

Rheumatoid arthritis (RA) is a complex and chronic autoimmune disease characterized by joint inflammation and pain [25]. Our first case study utilizes the RA DNA methylation data from Liu *et al.* [26], which includes data profiled using the Infinium HumanMethylation450 BeadChip on 689 participants (335 controls and 354 RA cases), obtained from GEO (accession number GSE42861).

To ensure numerical stability, a threshold of $\epsilon = 10^{-6}$ was applied, replacing all zero and one values in the methylation data with $\epsilon $ and $1-\epsilon $, respectively. We then normalized the data using the Beta-Mixture Quantile (BMIQ) method [19]. Probes with detection p-values$>10^{-16}$ were treated as missing values, and samples with a call rate below 95% as well as probes with a call rate below 90% were excluded. After filtering, 663 samples and 473,154 CpG sites remained. Additionally, two samples were excluded due to missing smoking status, resulting in a final dataset of 661 samples (323 controls and 338 RA cases). Missing methylation values were imputed using k=10 nearest neighbor imputation, based on the nearest neighbor averaging method [20, 21].

We then compared cell type proportions estimated using EpiDISH [27] with those estimated using Houseman’s method [28] from the minfi package. The Spearman rank correlation coefficients between EpiDISH and Houseman’s method for CD8T cells, CD4T cells, natural killer (NK) cells, B-cells, monocytes, and granulocytes were 0.765, 0.950, 0.913, 0.886, 0.819, and 0.976, respectively. Supplementary Fig. 31 provides the Spearman rank correlation coefficients stratified by disease status, showing that for each cell type, the correlation between the two methods is higher within the control group.

We compared each method with and without adjusting for covariates, including age, sex, and smoking status, using cell type proportions estimated from EpiDISH and Houseman’s method, respectively. In other words, for each method, four models were fitted: (a) EpiDISH cell proportions without covariate adjustment, (b) Houseman’s cell proportions without covariate adjustment, (c) EpiDISH cell proportions with covariate adjustment, and (d) Houseman’s cell proportions with covariate adjustment. Correlation heatmaps comparing the estimated negative log p-values for differential methylation tests (case versus control) across these methods are provided in Supplementary Figs 32–36.

Overall, the models with and without covariate adjustment showed high correlations across most methods, consistent with the study design, as the control group in this dataset was matched for age, sex, and smoking status. However, the results were sensitive to the algorithm used to estimate cell type proportions. Notably, differential methylation analysis within CD8T cells showed the lowest correlation between EpiDISH and Houseman across all five methods. In contrast, differential methylation analyses within CD4T cells, monocytes, and granulocytes showed the highest correlation between EpiDISH and Houseman, with the exception of the HIRE method.

While we initialized HIRE using the cell type estimates from EpiDISH and Houseman, re-estimation via a generalized expectation-maximization algorithm yielded cell type proportions with reduced correlation to the original estimates, especially for B-cells and monocytes (Supplementary Fig. 37). This led to significantly lower correlations in cell-type-specific differential methylation analyses between the initial cell type estimates. For NK cells, the correlation between EpiDISH and Houseman was moderate, with an average Spearman rank correlation ($\hat{r}$) of 0.47 across CellDMC, TCA, TOAST, and CeDAR. For B-cells, correlations remained high for CellDMC, TOAST, and CeDAR, but were lower for TCA ($\hat{r}=0.41$).

Additionally, CeDAR exhibited the highest correlations across all cell types when comparing cell-type-specific differential methylation analysis using estimates from EpiDISH versus Houseman. In the subsequent analysis, we report results using cell type proportions estimated with Houseman’s method, consistent with Liu *et al.* [26], and without covariate adjustment.

**Figure 6:**
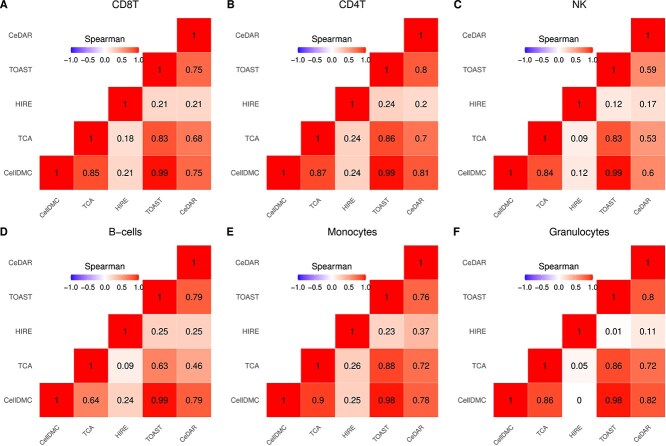
Correlation heatmap comparing the five methods within each cell type in rheumatoid arthritis case study in rheumatoid arthritis case study. Spearman rank correlation coefficients are provided in each cell.

Figure 6 presents correlation heatmaps comparing the five methods for cell-type-specific differential methylation analysis. The results from CellDMC and TOAST exhibit the highest correlations across cell types, as both methods use a linear model approach. TCA also shows high correlation with CellDMC and TOAST across all cell types except B-cells ($\hat{r}=0.64$)). The correlations between CeDAR and CellDMC, as well as TOAST, are above 0.75 for all cell types except NK cells ($\hat{r}\approx 0.6$). In contrast, HIRE shows the lowest correlation with other methods across all cell types.

**Figure 7:**
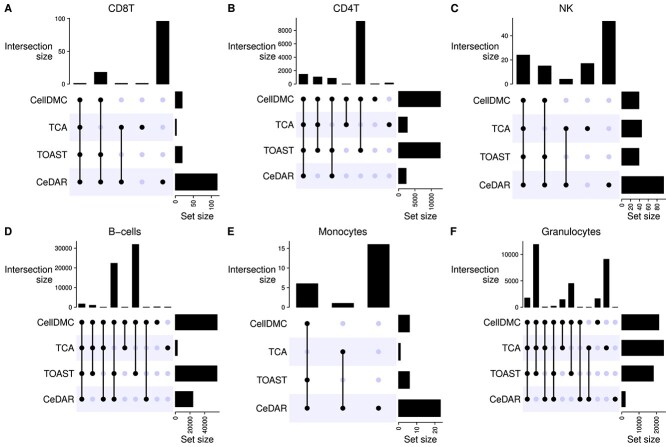
UpSet plot comparing the overlap among the four methods within each cell type in rheumatoid arthritis case study.

At FDR <0.01, Table 1 provides the number of CpGs identified by each method for each cell type. We further compared the overlaps among CellDMC, TCA, TOAST, and CeDAR via UpSet plots in Fig. 7. CellDMC and TOAST yield very similar numbers of significant CpGs across cell types, consistent with their high correlation observed in Fig. 6. TCA identifies fewer CpGs than CellDMC and TOAST, except in granulocytes. Among the five immune cell types (excluding granulocytes), CellDMC, TOAST, and CeDAR identify a greater number of CpGs in B-cells compared to other cell types. CeDAR also identifies fewer CpGs in CD4T, B-cells, and granulocytes compared to CellDMC and TOAST. The Venn diagram comparing all five methods including HIRE is provided in Supplementary Fig. 40, showing that HIRE identifies a substantially larger number of CpGs in CD8T cells, B-cells, and monocytes, many of which may be false positives, as suggested by inflated empirical FDRs in our simulation studies.

**Table 1:** Number of CpGs identified at FDR<0.01 in rheumatoid arthritis case study

	CD8T	CD4T	NK	B-cells	Mono	Gran
CellDMC	19	12768	39	57079	6	21481
TCA	3	2677	45	2755	1	24230
HIRE	11500	1563	3951	138528	252200	42886
TOAST	19	12736	39	56873	6	18344
CeDAR	116	2320	95	24010	23	2024
$avepv$	1	5380	14	28871	0	11261
$minpv$	8	8932	34	49434	7	17295

When comparing the overlaps among these methods, CpGs identified by CellDMC and TOAST completely overlap across all cell types. The overlap between CellDMC and CeDAR averages around 99%, while the overlap between TCA and CellDMC, as well as CeDAR, averages around 59% and 81%, respectively. HIRE shows much lower overlap, averaging 33% with CellDMC, 24% with TCA, and 42% with CeDAR. Given the high correlation between CellDMC and TOAST and the poorer performance of HIRE across various metrics, we combined the results from CellDMC, TCA, and CeDAR using the $minpv$ and $avepv$ methods. The number of CpGs identified by these combined approaches is presented in the last two rows of Table 1, reflecting the trends observed in the simulation studies. The $avepv$ method tends to be more conservative, while the $minpv$ approach strikes a balance, identifying a comparable number of significant CpGs to the individual methods. The overlap of $minp$v with CellDMC, TCA, and CeDAR is 88%, 93%, and 97%, respectively.

Supplementary Table 1 compares the 10 differentially methylated CpGs in B-cells associated with RA, as reported by Julia *et al.* [29], with the results from our study. CellDMC, HIRE, TOAST, minpv, and avepv identify 6 of these CpGs, while CeDAR identifies 5, and TCA does not identify any. We also compare the ranks of these 10 CpGs according to p-values from B cell-type-specific differential methylation analysis for each method in Supplementary Table 1. The $avepv$ method achieves the lowest median rank (18,497.5 out of 473,154 CpGs), followed by $minpv$, TOAST, CellDMC, and CeDAR, which have comparable median ranks. HIRE and TCA yield median ranks of 85,205 and 185,191, respectively, indicating that these two methods perform worse compared to others in validating B cell-type-specific CpGs associated with RA.

Next, we performed gene set analysis on the ranked list of CpGs, based on p-values, using methylGSA [30], which adjusts for gene length, i.e. the number of CpGs per gene [31], for KEGG and GO databases. We set the minimum and maximum sizes for the gene sets to 50 and 500, resulting in 122 KEGG and 3,466 GO gene sets tested. First, we compared the correlations of the negative log p-values for the KEGG and GO gene sets from the gene set analysis results (Supplementary Figs 38 and 39). The correlation heatmaps show that CellDMC, TOAST, and CeDAR are highly correlated in both KEGG and GO gene set analyses, whereas HIRE shows lower correlations with the other methods.

Using FDR<0.01, we identified significant KEGG and GO gene sets. We further applied REVIGO [32] to identify representative subsets of GO terms. Supplementary Tables 2 and 3 summarize the number of KEGG and GO gene sets identified by each method within each cell type. All methods except HIRE identified the largest number of KEGG and GO gene sets in CD4T cells. Additionally, CellDMC, TOAST, CeDAR, $minpv$, and $avepv$ identified a large number of GO gene sets in B-cells.

Tables 2 and 3 list the representative KEGG and GO gene sets identified by the $minpv$ approach. “Cytokine-cytokine receptor interaction” was the top KEGG gene set associated with RA in CD8T cells, CD4T cells, and B-cells. This finding aligns with existing literature, which shows that cytokines regulate a wide range of inflammatory processes implicated in the pathogenesis of RA [33]. Likewise, immune- and inflammation-related gene sets were among the top GO gene sets identified (Table 3), consistent with the understanding that RA is an autoimmune disease characterized by joint inflammation [25]. Complete tables for each method can be found in Supplementary Tables 4 and 5.

**Table 2:** List of significant KEGG gene sets identified by the $minpv$ approach in RA case study

	Description
CD8T	-Cytokine-cytokine receptor interaction
	-Chemokine signaling pathway
	-Chagas disease
CD4T	-Cytokine-cytokine receptor interaction
	-Neuroactive ligand-receptor interaction
	-Cell adhesion molecules
	-Olfactory transduction
	-Hematopoietic cell lineage
	-Chemokine signaling pathway
	-Rheumatoid arthritis
	-Hypertrophic cardiomyopathy
	-Protein digestion and absorption
	-Systemic lupus erythematosus
	-Calcium signaling pathway
	-ECM-receptor interaction
	-Staphylococcus aureus infection
	-Dilated cardiomyopathy
	-Natural killer cell mediated cytotoxicity
B-cells	-Cytokine-cytokine receptor interaction
	-Olfactory transduction
	-Neuroactive ligand-receptor interaction
	-Pancreatic secretion
	-ECM-receptor interaction
	-Steroid hormone biosynthesis

**Table 3:** List of significant top representative GO gene sets identified by the $minpv$ approach in RA case study

	Description
CD8T	-Adaptive immune response based on somatic
	recombination of immune receptors built from
	immunoglobulin superfamily domains
	-Humoral immune response
	-Response to type II interferon
	-Positive regulation of receptor signaling pathway
	via JAK-STAT
	-Cellular response to biotic stimulus
	-Cellular response to lipopolysaccharide
	-Cellular response to type II interferon
	-T cell receptor complex
CD4T	-Cell chemotaxis
	-Humoral immune response
	-Cytokine activity
	-Intermediate filament organization
	-Positive regulation of ERK1 and ERK2 cascade
	-Chemokine-mediated signaling pathway
	-Phosphatidylinositol-mediated signaling
	-Keratinization
	-Postsynaptic membrane
	-Regulation of chemotaxis
NK	-Regulation of lamellipodium organization
	-Phosphatidylinositol bisphosphate phosphatase
	activity
B-cells	-Olfactory receptor activity
	-Detection of chemical stimulus involved in sensory
	perception of smell
	-Regionalization
	-Cell fate commitment
	-Extracellular structure organization
	-Extracellular matrix organization
	-External encapsulating structure organization
	-Glycosaminoglycan binding
	-Positive regulation of ERK1 and ERK2 cascade
	-Neuron fate commitment

### Case study 2

Major depressive disorder (MDD) is a mental health condition marked by a persistently low mood, diminished interest in activities, cognitive dysfunction, and physical symptoms such as sleep and appetite disturbances [34]. Our second case study utilizes MDD DNA methylation data from Mokhtari *et al.* [35], generated using the Infinium MethylationEPIC BeadChip on 147 participants (31 male controls, 27 male MDD cases, 50 female controls, and 39 female MDD cases), obtained from GEO (accession number GSE251780). We applied the same preprocessing steps described in the first case study. Given prior research indicating significant sex-specific differences in MDD biomarkers [36], we further filtered for CpGs on chromosomes X and Y. The final dataset comprises 146 samples (31 male controls, 26 male MDD cases, 50 female controls, and 39 female MDD cases) with data on 843,264 CpGs.

**Figure 8:**
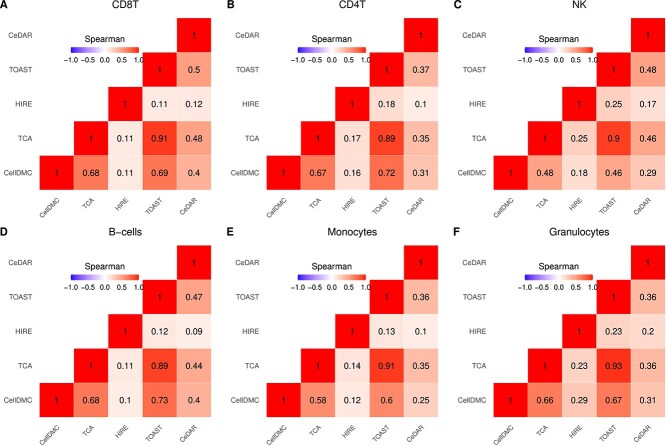
Correlation heatmap comparing the five methods within each cell type in analysis involving all participants in MDD case study. Spearman rank correlation coefficients are provided in each cell.

**Figure 9:**
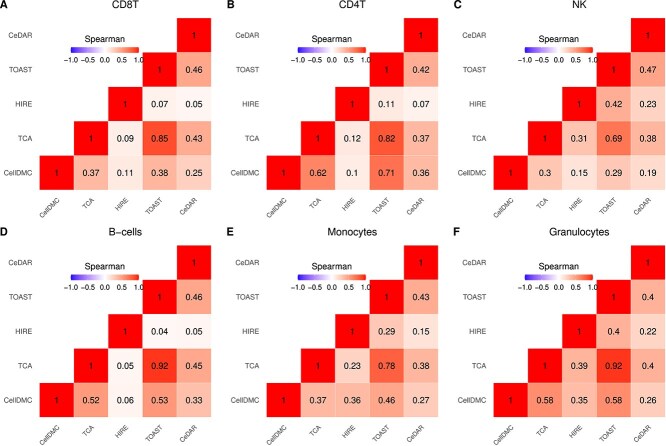
Correlation heatmap comparing the five methods within each cell type in analysis on subset of male participants in MDD case study. Spearman rank correlation coefficients are provided in each cell.

**Figure 10:**
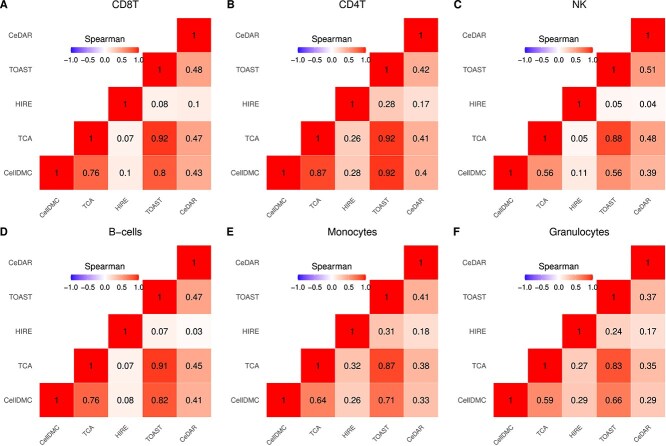
Correlation heatmap comparing the five methods within each cell type in analysis subset of female participants in MDD case study. Spearman rank correlation coefficients are provided in each cell.

Following Mokhtari *et al.* [35], we estimated cell type proportions using Houseman’s method. We then performed cell-type-specific differential methylation analysis on (a) all 146 participants, adjusting for age, sex, and BMI, and (b) male and female subsets separately, adjusting for age and BMI. Figures 8, 9, and 10 display correlation heatmaps comparing the five methods in cell-type-specific differential methylation analysis across all participants, as well as within male and female subsets, respectively. Among all methods, TCA and TOAST exhibit the highest correlations across all cell types, followed by CellDMC in comparison to TCA and TOAST within the female subset. Consistent with findings from the first case study, HIRE shows the lowest correlation with other methods across all cell types and subsets. Notably, correlations within the female subset are higher than those in the male subset, suggesting a stronger signal in females.

**Figure 11:**
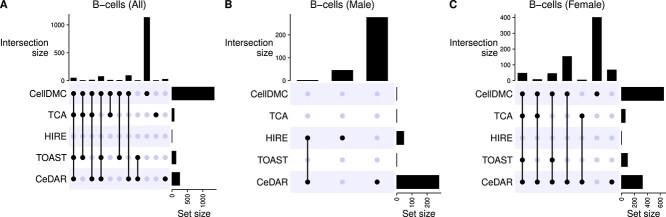
UpSet plot comparing the overlap among the five methods within B-cells in MDD case study.

We combined the results from CellDMC, TCA, TOAST, and CeDAR using the $minpv$ and $avepv$ methods. At FDR <0.05, Table 4 presents the number of significant CpGs identified by each method in each cell type across the three subset analyses. Across all methods (except for HIRE), the highest number of significant CpGs was detected in B-cells, with a stronger signal in the female subset compared to the male subset. For other cell types, only CeDAR identified cell-type-specific differentially methylated CpGs in both male and female subsets. At the epigenome-wide level, correlation heatmaps revealed a weak positive association between the male and female subsets in terms of differential methylation strength in MDD. This suggests notable sex differences in cell-type-specific differential methylation associated with MDD (Supplementary Figs 41–45). Additionally, the UpSet plots comparing the overlap among the five methods within B-cells (Fig. 11) reveal a lower degree of consensus. In contrast, the UpSet plots examining the overlap between male and female subsets for each cell type using CeDAR recapitulate observed sex differences (Supplementary Fig. 46).

**Table 4:** Number of CpGs identified at FDR<0.05 in MDD case study

All	CD8T	CD4T	NK	B-cells	Mono	Gran
CellDMC	0	0	31	1369	0	0
TCA	0	0	0	68	0	0
HIRE	0	1	3	0	0	39
TOAST	0	0	0	128	0	0
CeDAR	99	49	87	248	42	40
$avepv$	0	0	0	95	0	0
$minpv$	0	0	0	542	0	0
Male	CD8T	CD4T	NK	B-cells	Mono	Gran
CellDMC	0	2	0	0	0	0
TCA	0	0	0	0	0	0
HIRE	0	0	0	46	1	0
TOAST	0	0	0	0	0	0
CeDAR	239	208	210	277	188	201
$avepv$	0	0	0	0	0	0
$minpv$	0	0	0	1	0	0
Female	CD8T	CD4T	NK	B-cells	Mono	Gran
CellDMC	0	0	0	652	0	0
TCA	0	0	0	58	0	0
HIRE	3	1	14	0	0	0
TOAST	0	0	0	92	0	0
CeDAR	131	104	181	324	56	62
$avepv$	0	0	0	95	0	0
$minpv $	0	0	0	299	0	0

We conducted gene set analysis using methylGSA [30] to identify significant KEGG and GO gene sets. At FDR <0.1, the number of KEGG and GO gene sets identified for each method within each cell type across the three subset analyses is provided in Supplementary Tables 6-7. Overall, the results were weaker compared to the first case study on RA, likely due to the smaller sample sizes. Tables 5 and 6 present representative KEGG and GO gene sets identified using the $minpv$ approach for each subset analysis. Notably, the KEGG gene sets identified in the female subset analysis include “Systemic lupus erythematosus,” “Staphylococcus aureus infection,” and “Cytokine-cytokine receptor interaction.” These findings align with increasing evidence linking these pathways to MDD through mechanisms such as systemic inflammation, immune activation, hormonal imbalances, and gut microbiome interactions [37–40]. Similarly, the GO gene sets identified in the female subset analysis, including “Cysteine-type peptidase activity” and pathways related to ubiquitination and deubiquitination processes, are associated with MDD through their roles in synaptic transmission, neuroplasticity, and neuroinflammation [41, 42]. The stronger methylation signal observed in females compared to males may be attributed to factors such as heightened immune responses, hormonal influences, and increased inflammatory activity.

**Table 5:** List of significant KEGG gene sets identified by the $minpv$ approach in MDD case study. ** indicates FDR<0.1, * indicates p-value$<0.05$, - indicates not significant

	Description	All	Male	Female
CD8T	-Systemic lupus erythematosus	**	–	**
	-Staphylococcus aureus	*	–	**
	infection			
	-Cell adhesion molecules	*	*	**
NK	-ECM-receptor interaction	**	*	–
B-cells	-Cytokine-cytokine receptor	**	–	*
	interaction			
	-Staphylococcus aureus	**	–	**
	infection			
	-Cell adhesion molecules	*	–	**

**Table 6:** List of significant GO gene sets identified by the $minpv$ approach in MDD case study. ** indicates FDR<0.1, * indicates p-value$<0.05$, - indicates not significant

	Description	All	Male	Female
CD8T	-Response to osmotic stress	**	–	–
	-Purine nucleoside	**	–	–
	monophosphate metabolic			
	process			
	-Cellular response to osmotic	**	–	–
	stress			
	-Translation regulator activity,	**	–	–
	nucleic acid binding			
	-Acidic amino acid transport	**	–	*
	-Negative chemotaxis	–	**	–
CD4T	-Homophilic cell adhesion	–	–	**
	via plasma membrane			
	adhesion molecules			
B-cells	-Neurotransmitter receptor	–	**	–
	complex			
	-Regulation of reactive oxygen	*	–	**
	species biosynthetic process			
Mono	-B cell activation involved	–	**	
	in immune response			
	-Regulation of heart rate	–	–	**
	by cardiac conduction			
	-Cysteine-type deubiquitinase	*	–	**
	activity			
	-Ubiquitin-like protein	*	–	**
	peptidase activity			
Gran	-Cysteine-type deubiquitinase	*	–	**
	activity			
	-Ubiquitin-like protein	*	–	**
	peptidase activity			
	-Protein deubiquitination	*	–	**
	-Cysteine-type peptidase	–	–	**
	activity			
	-Organic hydroxy compound	–	–	**
	transmembrane transporter			
	activity			

## Discussion

Cell-type-specific differential methylation is crucial because DNA methylation, a key epigenetic mechanism, can vary between different cell types within the same tissue. Studying these variations provides critical insights into biological processes and disease mechanisms, improving biomarker discovery and identifying therapeutic targets. Recent advances in computational models for DNA methylation analysis have led to the development of specialized cell-type-specific differential methylation models for bulk tissues. In this paper, we systematically evaluated several of these models through simulations and a case study to assess their strengths and limitations.

Our comprehensive evaluation reveals significant variability in performance across different metrics and computational efficiency. Notably, CeDAR achieved the highest AUROC across all cell types and SNR levels in the simulation study. However, CeDAR’s sensitivity decreased with smaller cell proportions, whereas CellDMC maintained consistent performance across varying cell proportions. TCA and TOAST showed comparable performance metrics, while HIRE exhibited an elevated FDR in the simulation study. All methods experienced performance declines as the SNR decreased, underscoring the challenge of maintaining accuracy under noisier conditions.

In the case studies, where cell type proportions were estimated, we observed that the accuracy of the estimated cell proportions influenced the results of these methods. Accurate cell type proportion estimation is a critical prerequisite for cell-type-specific differential methylation analysis, as supported by our previous findings, which demonstrated the importance of using appropriate reference libraries for accurate deconvolution algorithms in estimating cell proportions [43]. TOAST emerged as the most efficient algorithm, followed by CellDMC, making them ideal for large-scale studies where computational resources and time are limited. HIRE had the slowest runtime among the algorithms.

The substantial overlap in differentially methylated CpGs detected by the various models in the RA case study, except for HIRE, suggests some consensus across these methods when the sample size is large. However, the unique CpGs identified by each model underscore the potential for complementary insights when multiple methods are used. Conversely, the lower degree of consensus among the models in the MDD case study highlights the importance of sample size in improving the accuracy of cell-type-specific differential methylation analysis. Our proposed $minpv$ and $avepv$ approaches further demonstrate the benefits of model integration, offering a more robust and comprehensive understanding of methylation changes and yielding more accurate conclusions in epigenomic research. Specifically, the $minpv$ approach showed superior performance across all metrics in the simulation study, while the $avepv$ approach, though more conservative, achieved the highest AUROC and the lowest median rank in detecting validated B-cell-type-specific CpGs in the case study.

As more methods are developed, we anticipate that both the $minpv$ and $avepv$ approaches will continue to enhance the accuracy of cell-type-specific differential methylation analysis by integrating the strengths of multiple methods while balancing their limitations.

**Key Points**

- Cell-type-specific differential methylation is crucial for understanding biological processes and disease mechanisms, as DNA methylation can differ between various cell types within the same tissue.
- We systematically evaluated five cell-type-specific differential methylation models on bulk data: CellDMC, TCA, HIRE, TOAST, and CeDAR.
- The results showed that the models differed in performance across various metrics, sample sizes, and computational efficiency.
- Our proposed method, which aggregates results using the minimum p-value approach, outperforms the individual models.

## Supplementary Material

SM_CellSpecDMC_v4a_revision_noMarking_bbaf170

## Data Availability

The data underlying this article are available in the Gene Expression Omnibus (GEO, https://www.ncbi.nlm.nih.gov/geo) and the EMBL’s European Bioinformatics Institute (https://www.ebi.ac.uk), and can be accessed with accession numbers GSE31848, GSE59250, GSE71244, GSE50222, GSE56047, GSE42861, GSE110554, GSE251780, and E-MTAB-2145 (EBI), respectively.

## Code availability

A pipeline implementing our proposed $minpv$ and $avepv$ approaches for integrating results from cell-type-specific differential methylation algorithms is available at https://github.com/Leesure001/Integrative_CTS_DNAm.
