# Supplementary material for: A systematic evaluation of cell-type-specific differential methylation analysis in bulk tissue
Source: Brief Bioinform. 2025 Apr 16;26(2):bbaf170. doi: 10.1093/bib/bbaf170 (PMC12001786; doi:10.1093/bib/bbaf170)
Supplement: SM_CellSpecDMC_v4a_revision_noMarking_bbaf170 [file sm_cellspecdmc_v4a_revision_nomarking_bbaf170.pdf]

Supplementary Materials: A systematic evaluation of cell-type-specific differential methylation analysis in bulk tissue

Shuo Li
Department of Applied Mathematics and Statistics,
Stony Brook University,
Stony Brook, NY 11794

Pei Fen Kuan*
Department of Applied Mathematics and Statistics,
Stony Brook University,
Stony Brook, NY 11794

*Corresponding Author: peifen.kuan@stonybrook.edu

Supplementary Figures

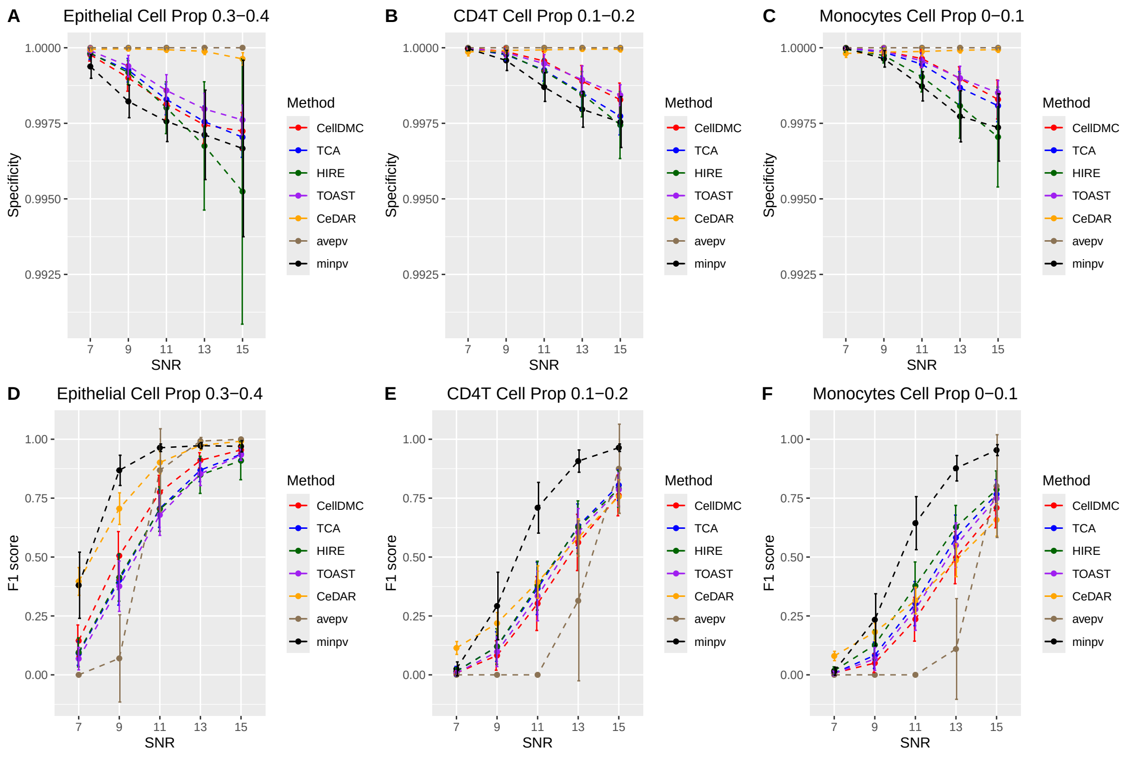

Figure S1: Specificity and F1 score across different methods and cell type proportions for $n = 100$ based on Illumina 450k benchmarking datasets. A-C. Specificity plots for cell-type-specific differential methylation analysis in epithelial, CD4T and monocytes, respectively. D-F. F1 score plots.

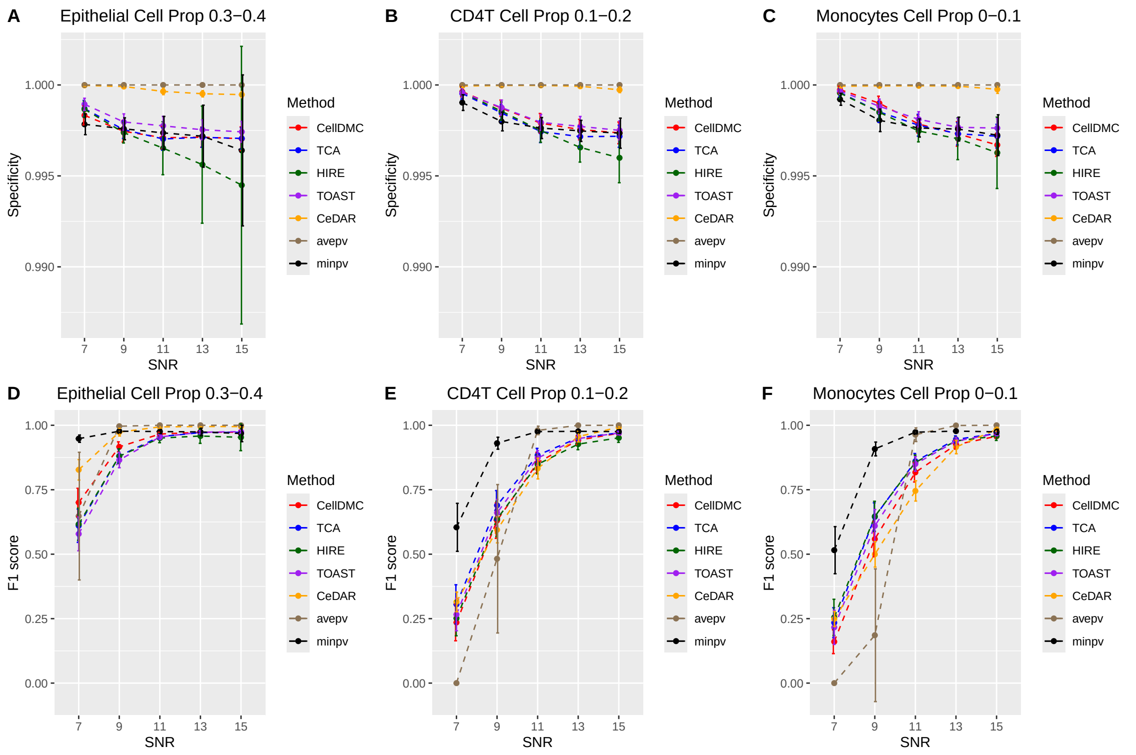

Figure S2: Specificity and F1 score across different methods and cell type proportions for $n = 200$ based on Illumina 450k benchmarking datasets. A-C. Specificity plots for cell-type-specific differential methylation analysis in epithelial, CD4T and monocytes, respectively. D-F. F1 score plots.

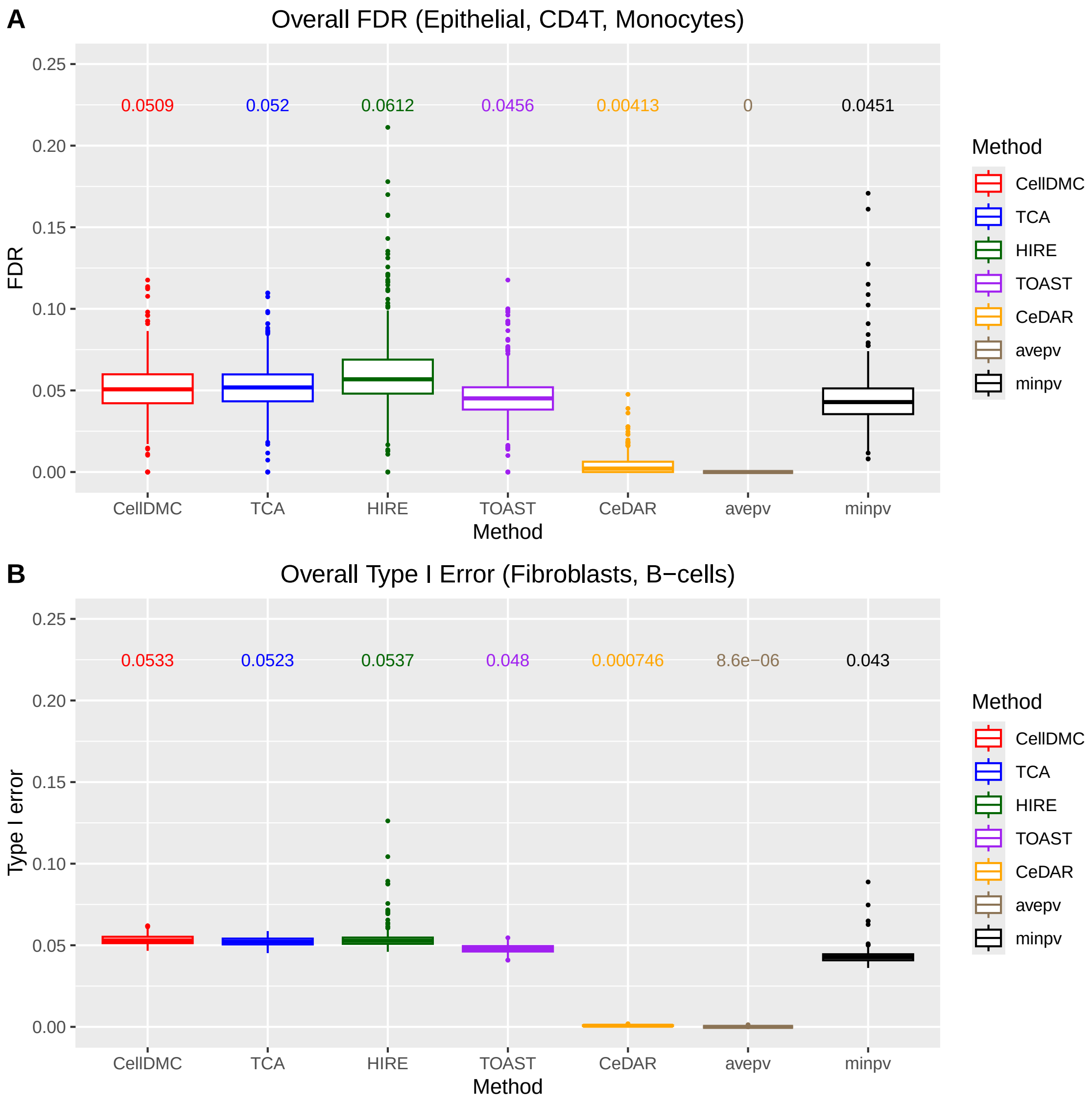

Figure S3: A. Boxplots of empirical FDR across different methods aggregating results for for cell-type-specific differential methylation analysis in epithelial, CD4T and monocytes, and SNRs for $n = 200$ based on Illumina 450k benchmarking datasets. B . Boxplots of empirical type I error across different methods aggregating results for cell-type-specific differential methylation analysis in fibroblasts and B-cells, and SNRs for $n = 200$ based on Illumina 450k benchmarking datasets. The printed numbers are the mean values of each method.

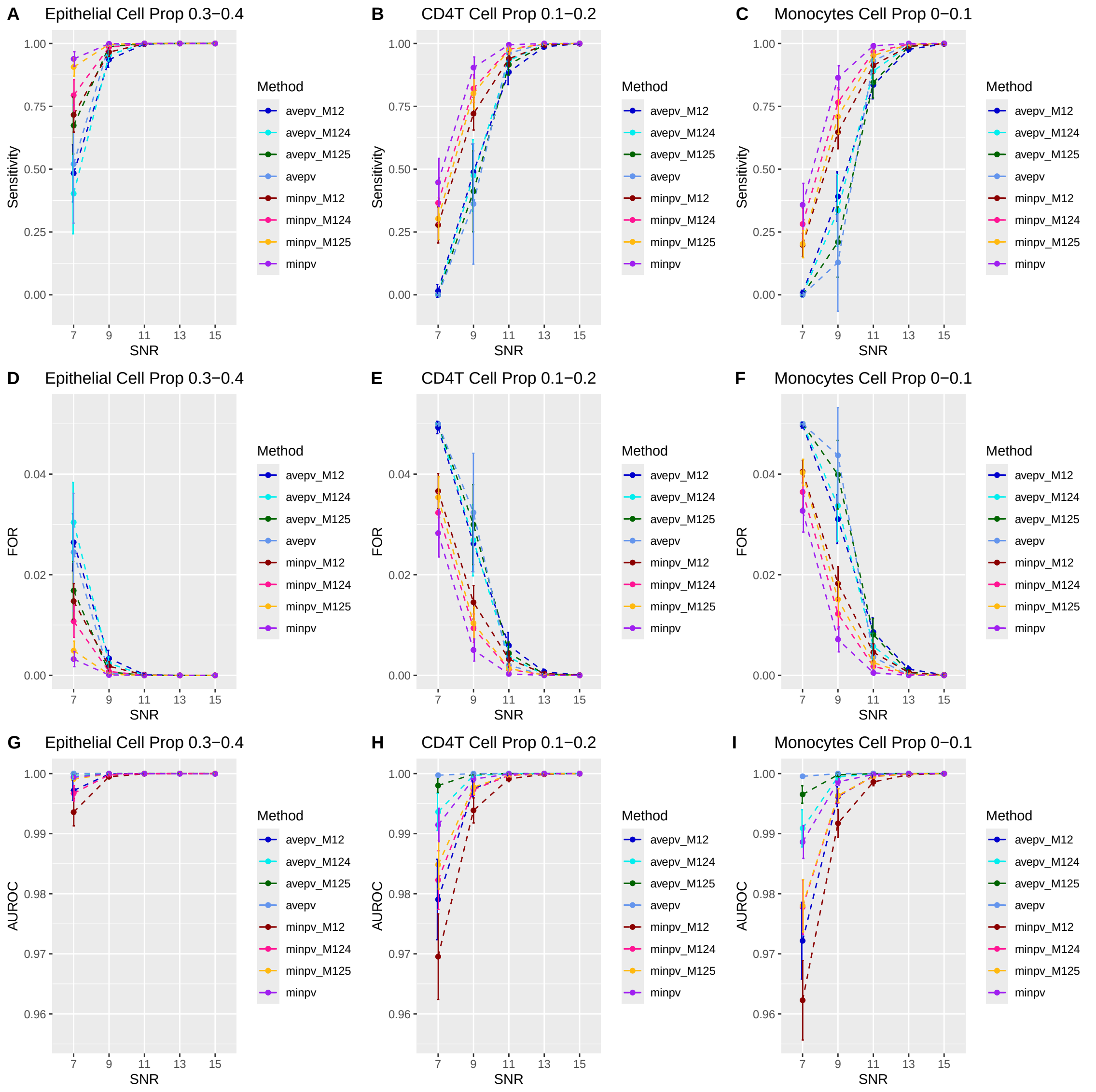

Figure S4: Sensitivity, FOR and AUROC across different result aggregation methods and cell type proportions for $n = 200$ based on Illumina 450k benchmarking datasets (minpv and avepv refer to aggregating results from all five methods). A-C. Sensitivity plots for cell-type-specific differential methylation analysis in epithelial, CD4T and monocytes, respectively. D-F. FOR plots. G-I. AUROC plots.

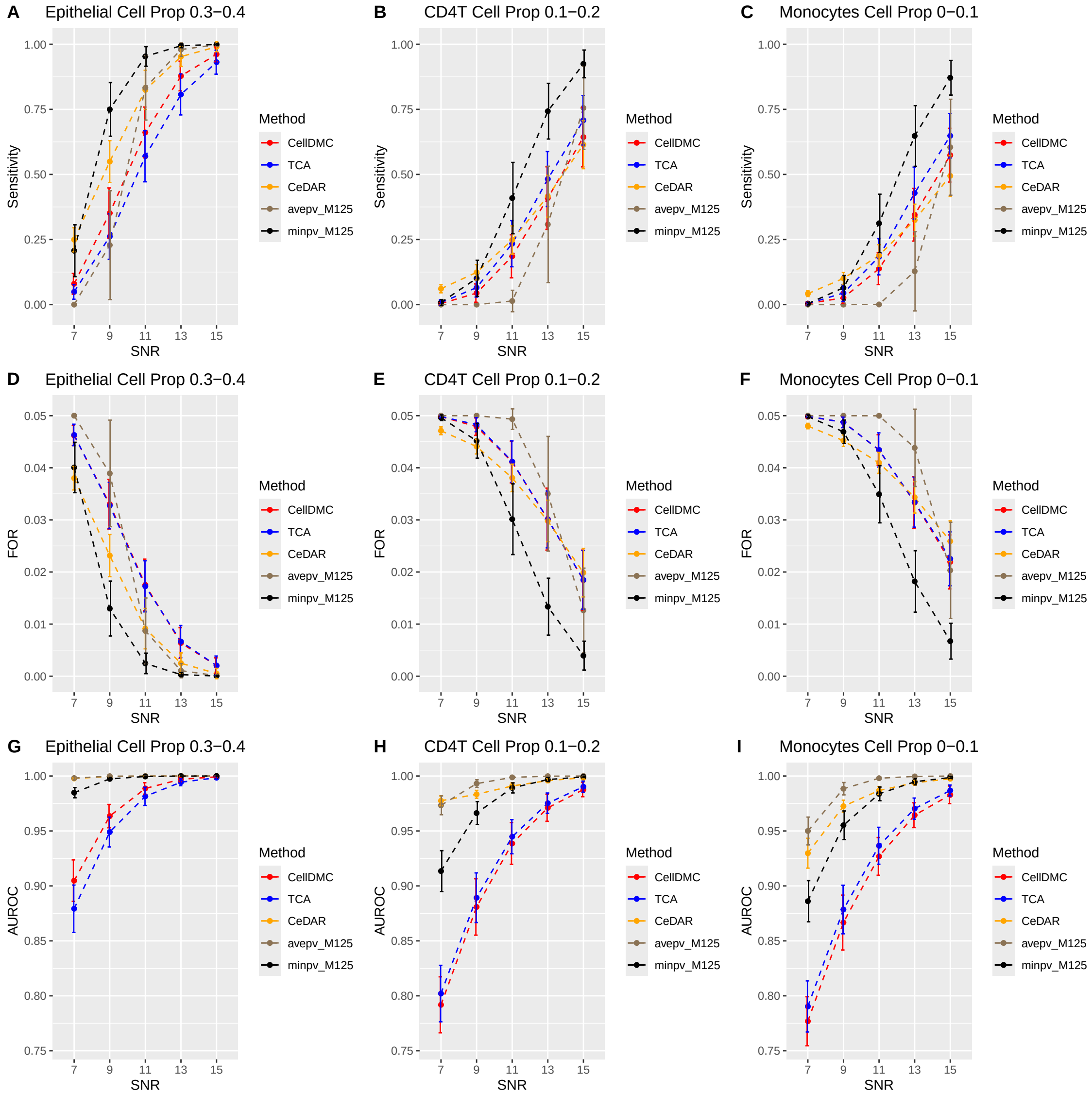

Figure S5: Sensitivity, FOR and AUROC across different result aggregation methods and cell type proportions for $n = 100$ comparing CellDMC, TCA, CeDAR and result aggregation methods which combine these three methods based on Illumina 450k benchmarking datasets. A-C. Sensitivity plots for cell-type-specific differential methylation analysis in epithelial, CD4T and monocytes, respectively. D-F. FOR plots. G-I. AUROC plots.

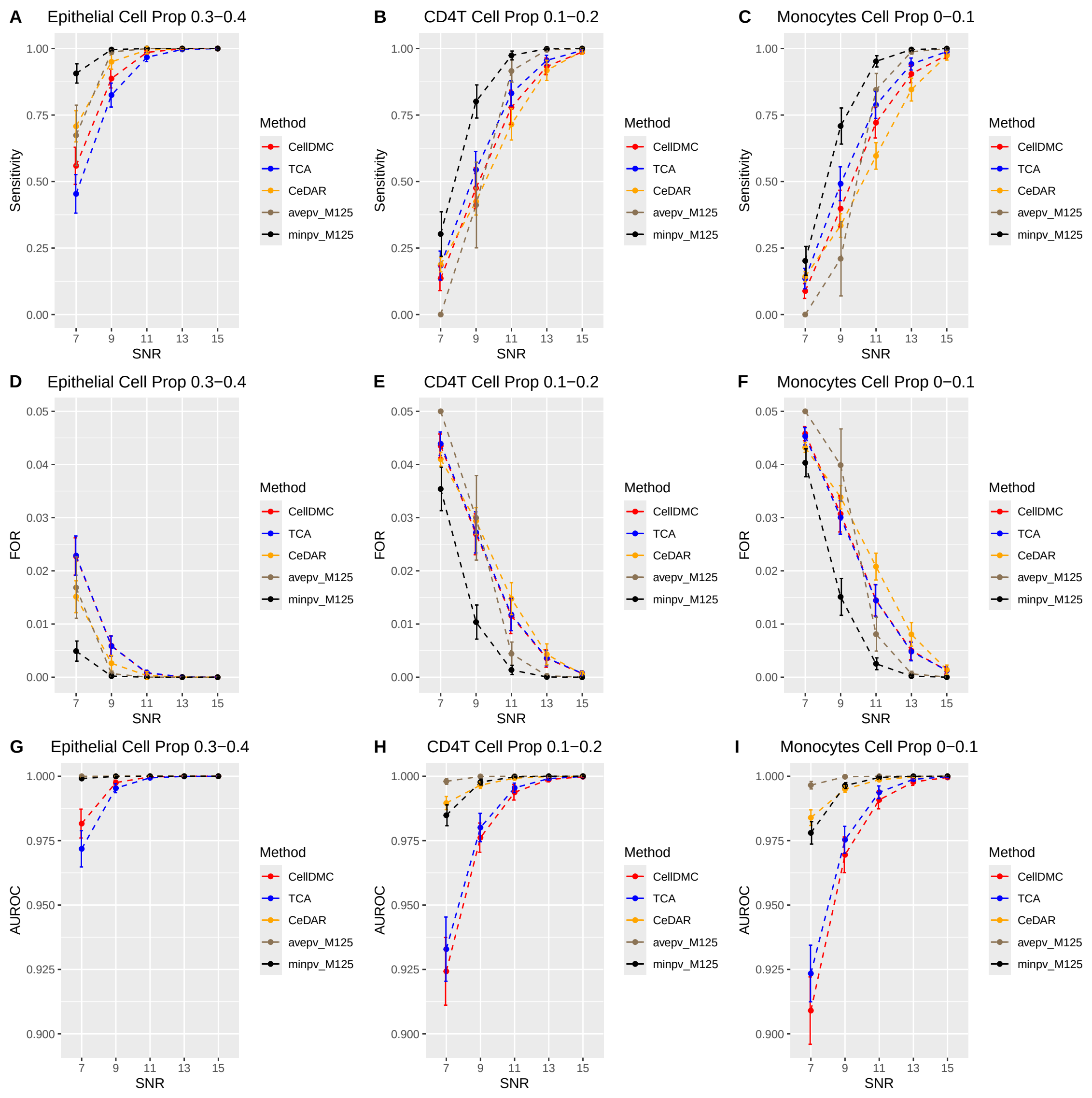

Figure S6: Sensitivity, FOR and AUROC across different result aggregation methods and cell type proportions for $n = 200$ comparing CellDMC, TCA, CeDAR and result aggregation methods which combine these three methods based on Illumina 450k benchmarking datasets. A-C. Sensitivity plots for cell-type-specific differential methylation analysis in epithelial, CD4T and monocytes, respectively. D-F. FOR plots. G-I. AUROC plots.

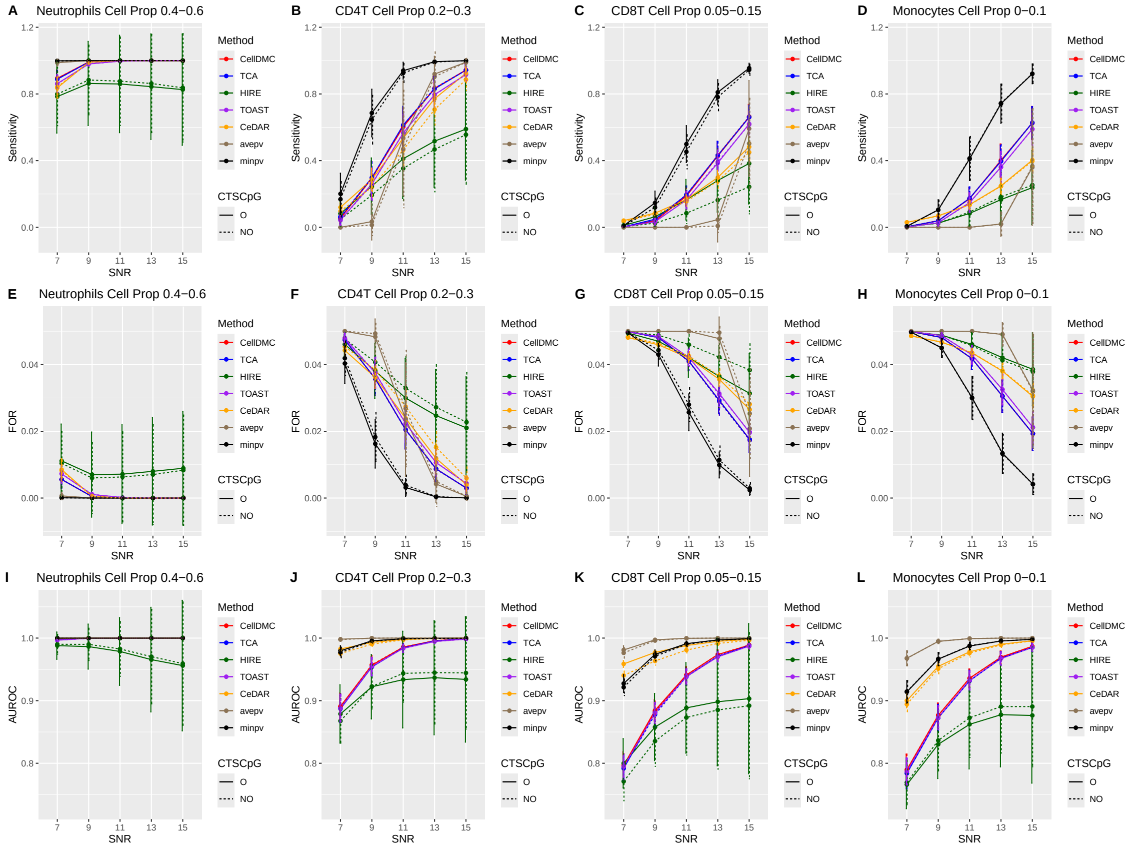

Figure S7: Sensitivity, FOR and AUROC across different methods and cell type proportions for $n = 100$ based on Illumina MethylationEPIC benchmarking datasets in Settings 1a (NO: no overlapping cell-type-specific effect CpGs) and 1b (O: overlapping cell-type-specific effect CpGs in CD4T and CD8T). A-C. Sensitivity plots for cell-type-specific differential methylation analysis in neutrophils, CD4T, CD8T and monocytes, respectively. D-F. FOR plots. G-I. AUROC plots.

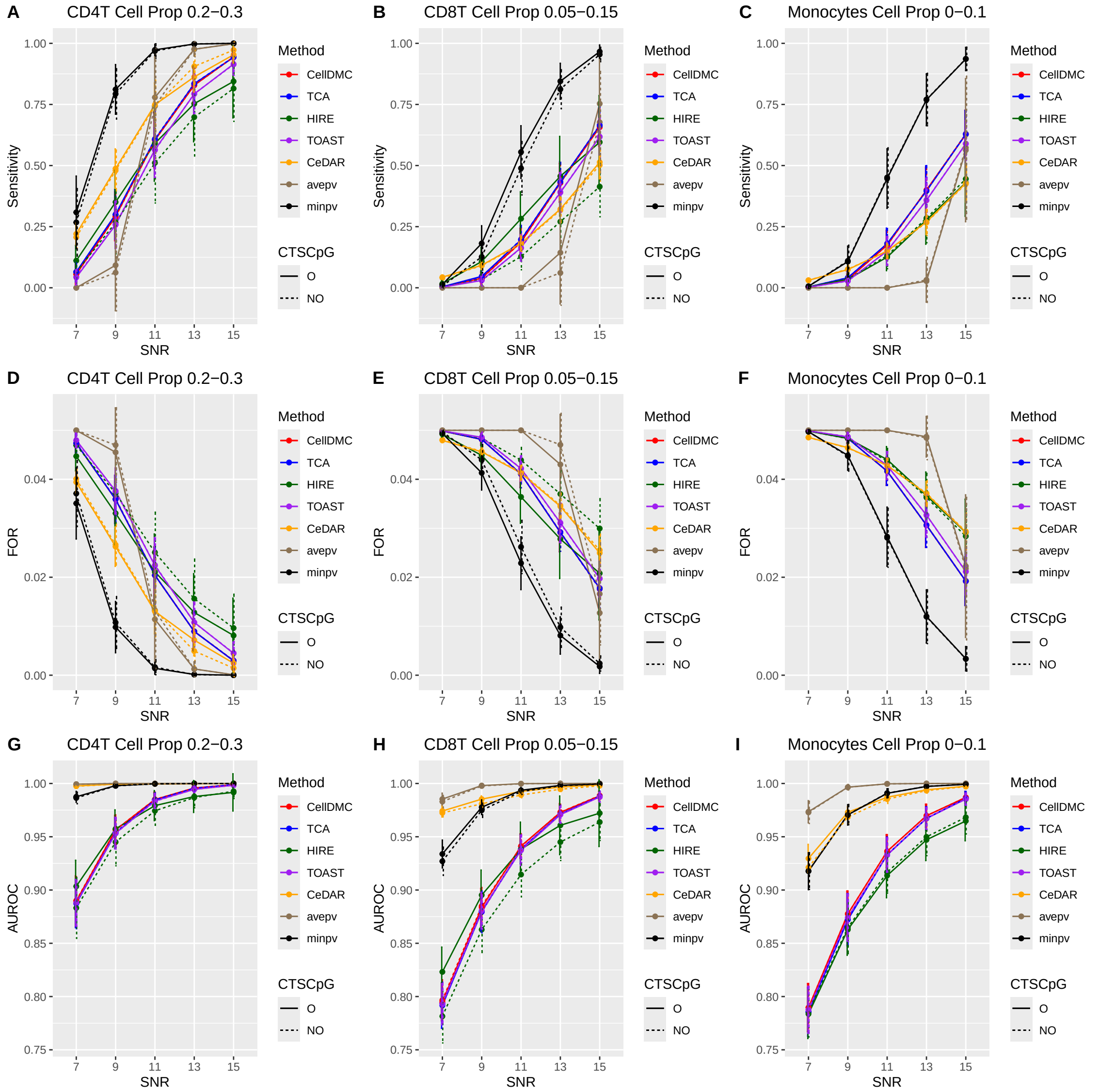

Figure S8: Sensitivity, FOR and AUROC across different methods and cell type proportions for $n = 100$ based on Illumina MethylationEPIC benchmarking datasets in Settings 2a (NO: no overlapping cell-type-specific effect CpGs) and 2b (O: overlapping cell-type-specific effect CpGs in CD4T and CD8T). A-C. Sensitivity plots for cell-type-specific differential methylation analysis in CD4T, CD8T and monocytes, respectively. D-F. FOR plots. G-I. AUROC plots.

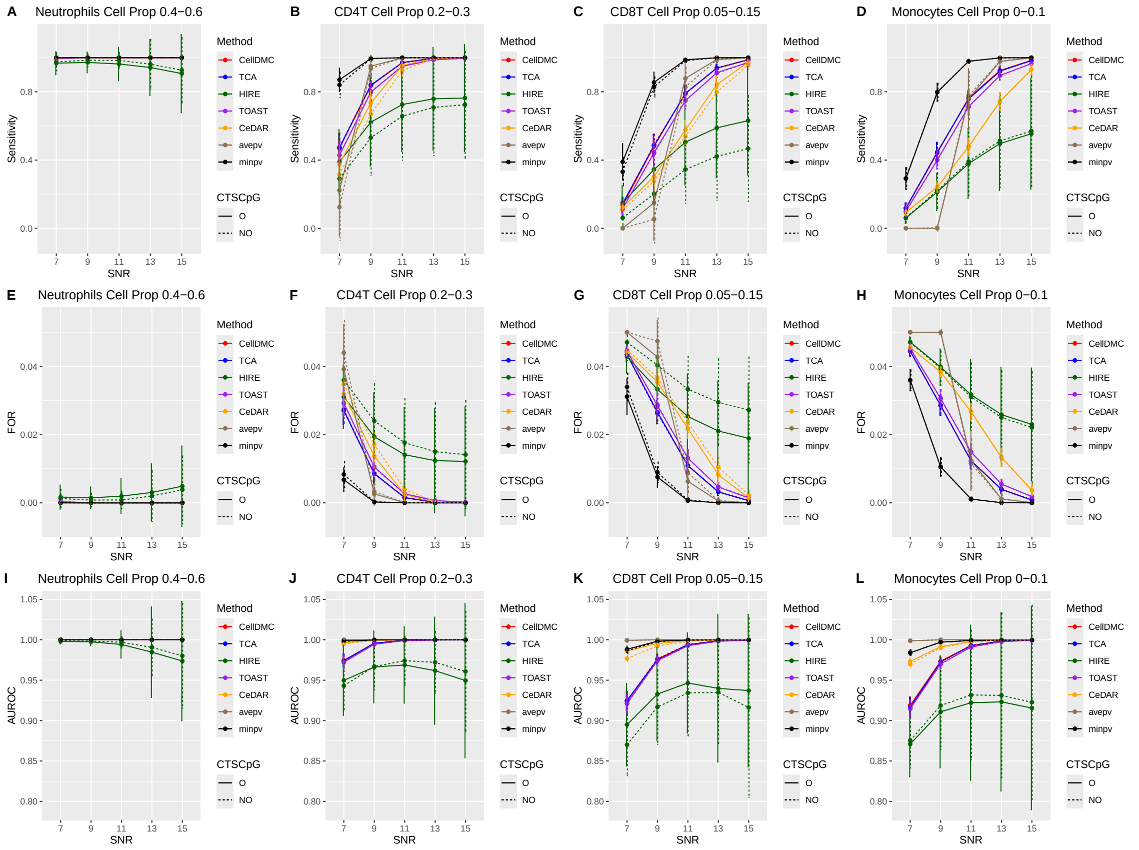

Figure S9: Sensitivity, FOR and AUROC across different methods and cell type proportions for $n = 200$ based on Illumina MethylationEPIC benchmarking datasets in Settings 1a (NO: no overlapping cell-type-specific effect CpGs) and 1b (O: overlapping cell-type-specific effect CpGs in CD4T and CD8T). A-C. Sensitivity plots for cell-type-specific differential methylation analysis in neutrophils, CD4T, CD8T and monocytes, respectively. D-F. FOR plots. G-I. AUROC plots.

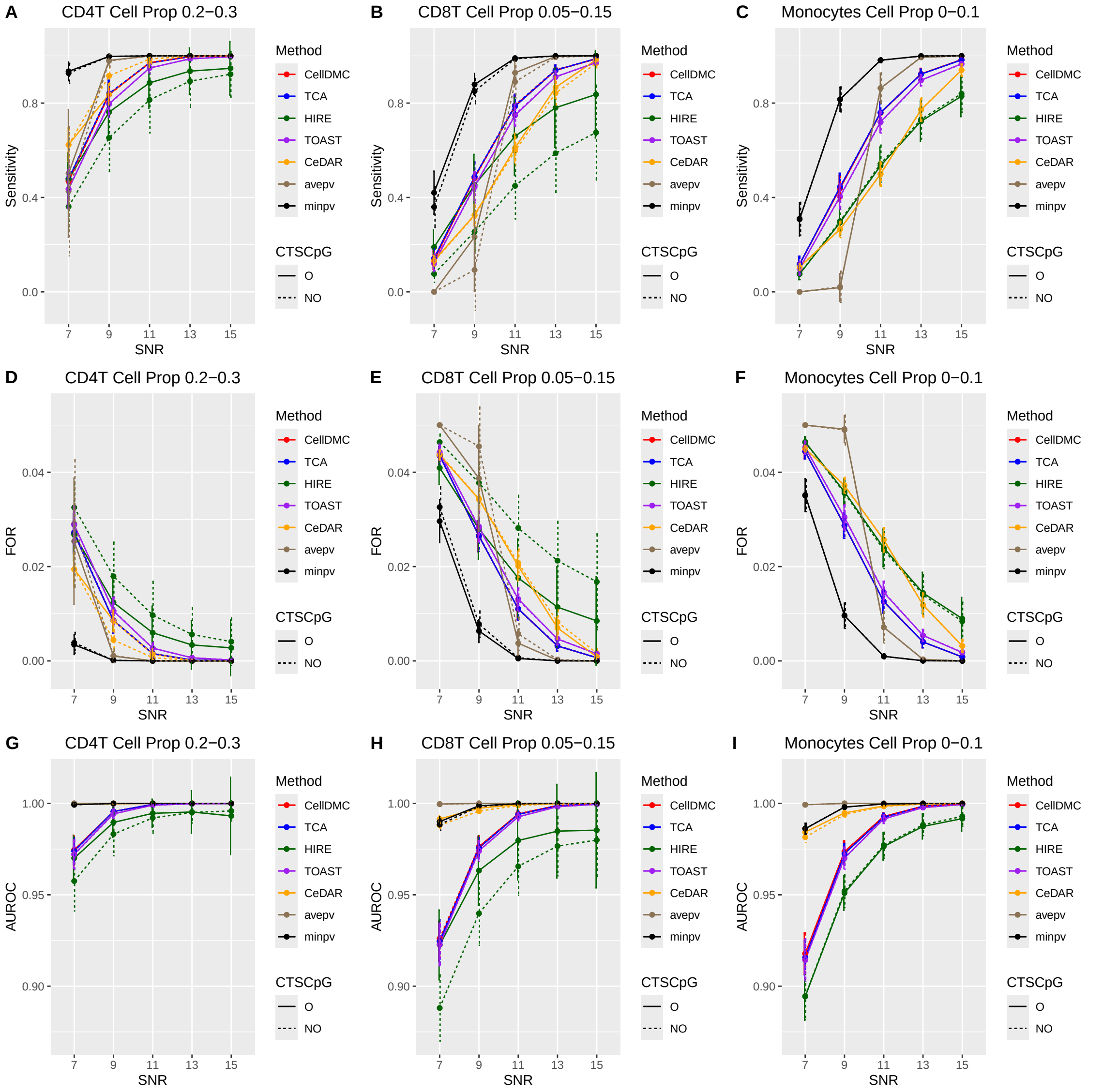

Figure S10: Sensitivity, FOR and AUROC across different methods and cell type proportions for $n = 200$ based on Illumina MethylationEPIC benchmarking datasets in Settings 2a (NO: no overlapping cell-type-specific effect CpGs) and 2b (O: overlapping cell-type-specific effect CpGs in CD4T and CD8T). A-C. Sensitivity plots for cell-type-specific differential methylation analysis in CD4T, CD8T and monocytes, respectively. D-F. FOR plots. G-I. AUROC plots.

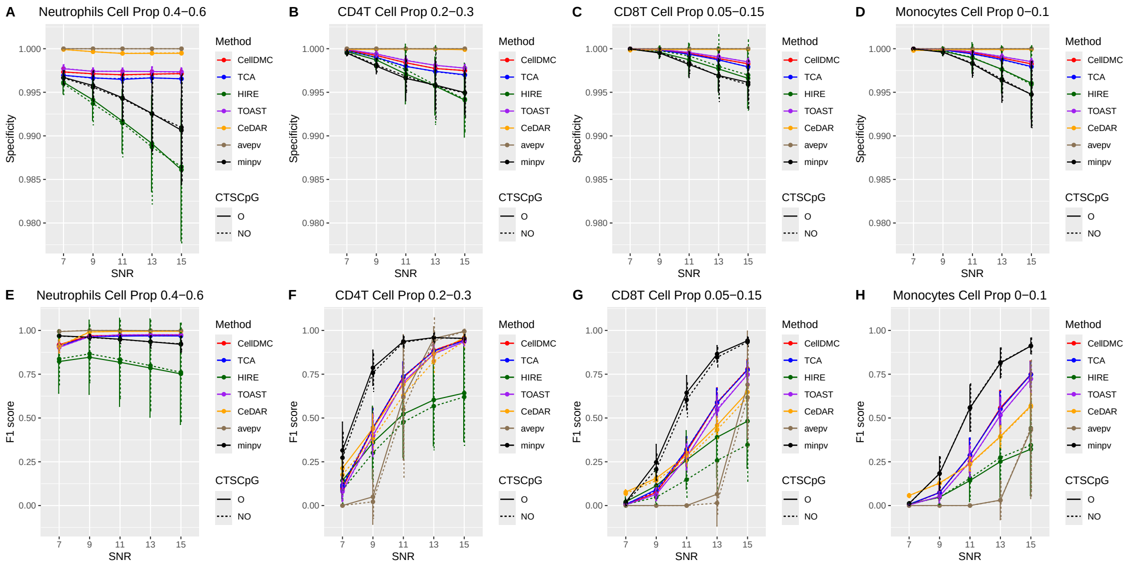

Figure S11: Specificity and F1 score across different methods and cell type proportions for $n = 100$ based on Illumina MethylationEPIC benchmarking datasets in Settings 1a (NO: no overlapping cell-type-specific effect CpGs) and 1b (O: overlapping cell-type-specific effect CpGs in CD4T and CD8T). A-C. Specificity plots for cell-type-specific differential methylation analysis in neutrophils, CD4T, CD8T and monocytes, respectively. D-F. F1 score plots.

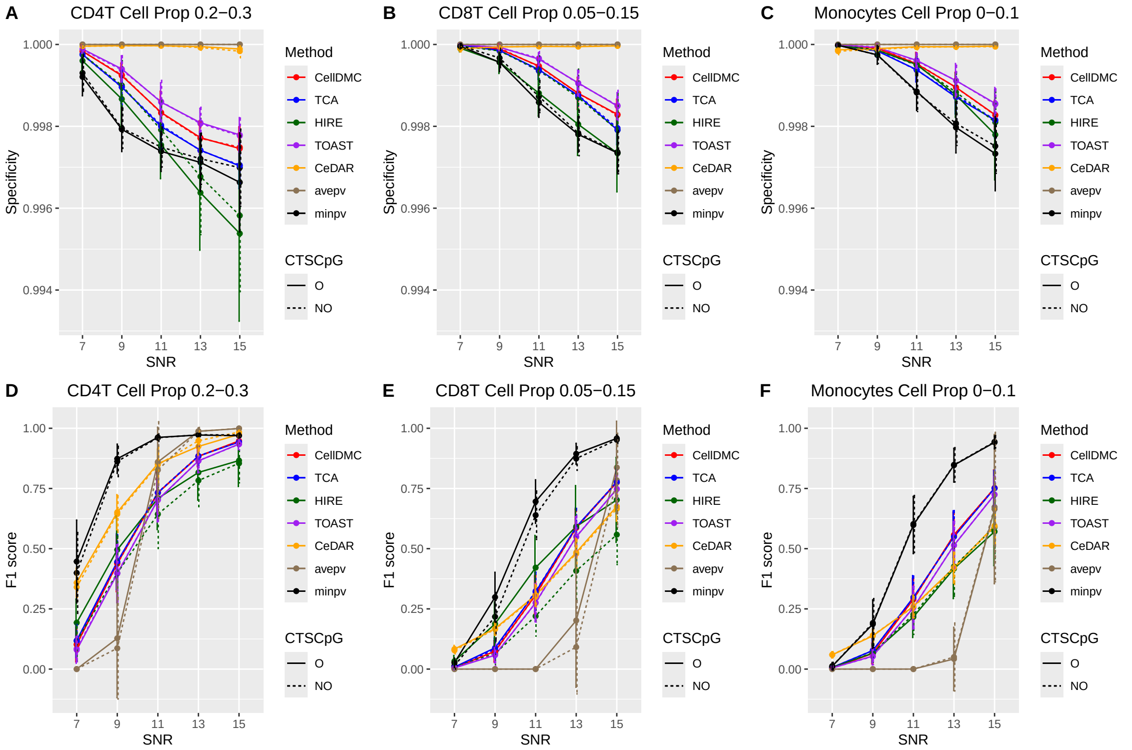

Figure S12: Specificity and F1 score across different methods and cell type proportions for $n = 100$ based on Illumina MethylationEPIC benchmarking datasets in Setting Settings 2a (NO: no overlapping cell-type-specific effect CpGs) and 2b (O: overlapping cell-type-specific effect CpGs in CD4T and CD8T). A-C. Specificity plots for cell-type-specific differential methylation analysis in CD4T, CD8T and monocytes, respectively. D-F. F1 score plots.

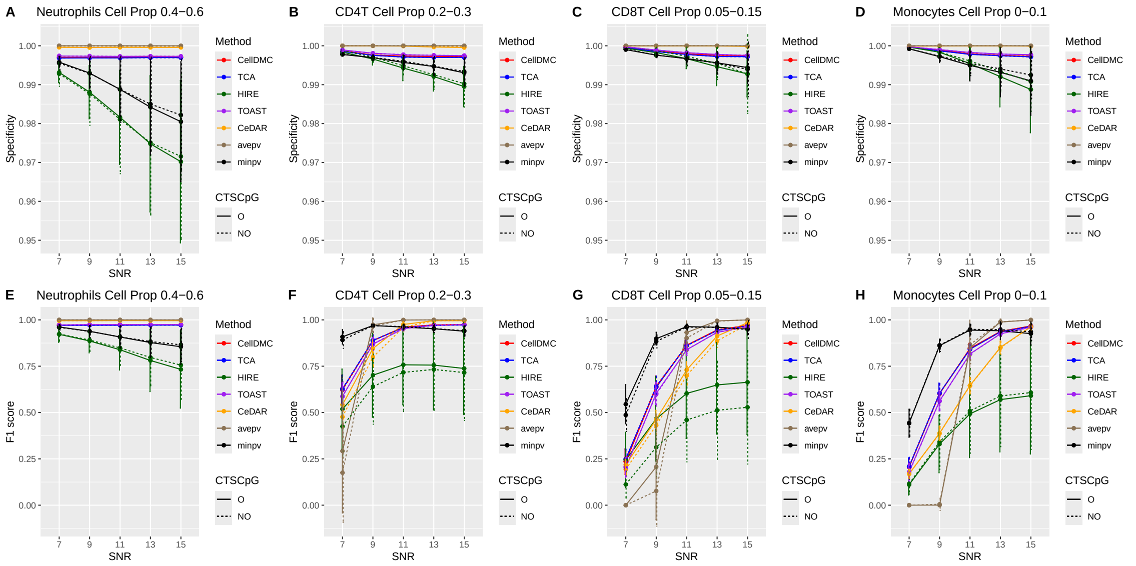

Figure S13: Specificity and F1 score across different methods and cell type proportions for $n = 200$ based on Illumina MethylationEPIC benchmarking datasets in Settings 1a (NO: no overlapping cell-type-specific effect CpGs) and 1b (O: overlapping cell-type-specific effect CpGs in CD4T and CD8T). A-C. Specificity plots for cell-type-specific differential methylation analysis in neutrophils, CD4T, CD8T and monocytes, respectively. D-F. F1 score plots.

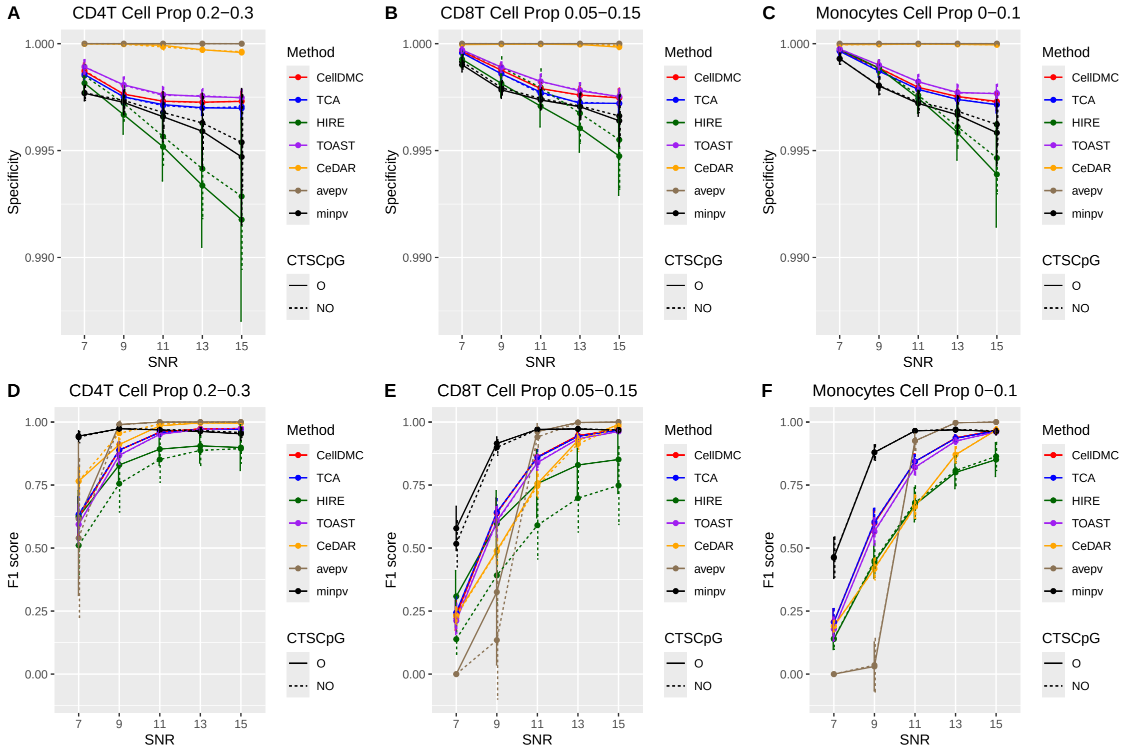

Figure S14: Specificity and F1 score across different methods and cell type proportions for $n = 200$ based on Illumina MethylationEPIC benchmarking datasets in Settings 2a (NO: no overlapping cell-type-specific effect CpGs) and 2b (O: overlapping cell-type-specific effect CpGs in CD4T and CD8T). A-C. Specificity plots for cell-type-specific differential methylation analysis in CD4T, CD8T and monocytes, respectively. D-F. F1 score plots.

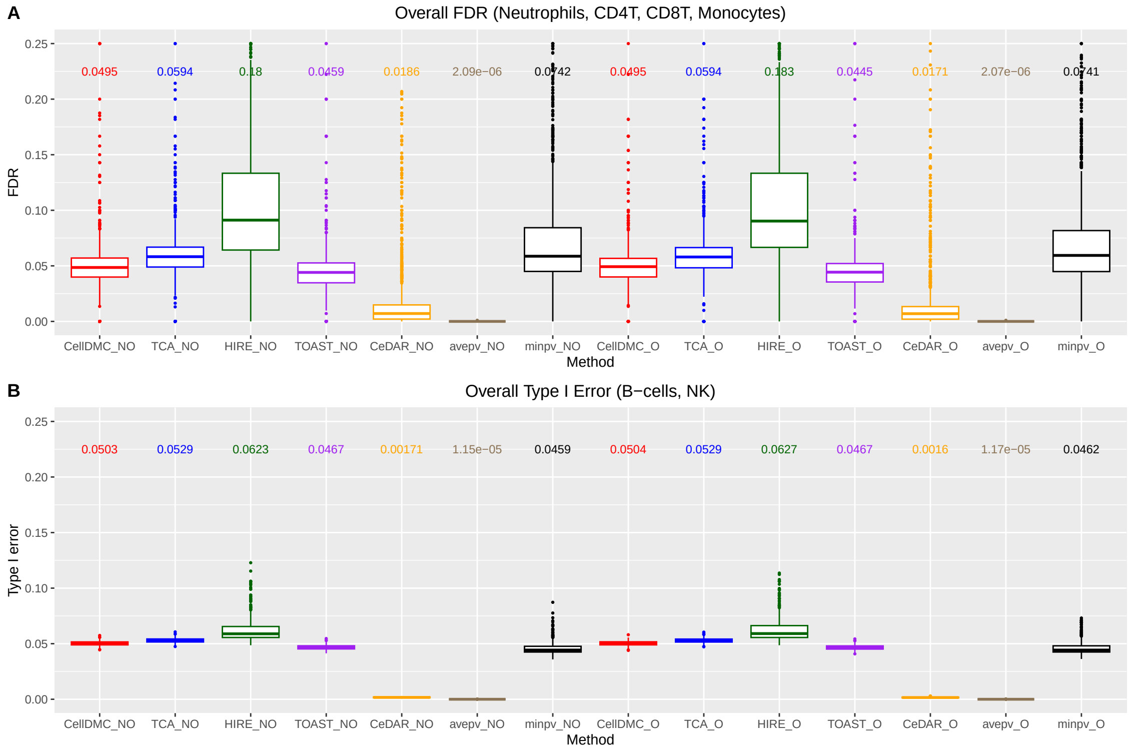

Figure S15: A. Boxplots of empirical FDR across different methods aggregating results for cell-type-specific differential methylation analysis in neutrophils, CD4T, CD8T and monocytes, and SNRs for $n = 100$ based on Illumina MethylationEPIC benchmarking datasets in Settings 1a (NO: no overlapping cell-type-specific effect CpGs) and 1b (O: overlapping cell-type-specific effect CpGs in CD4T and CD8T). B . Boxplots of empirical type I error across different methods aggregating results for cell-type-specific differential methylation analysis in B-cells and NK cells, and SNRs for $n = 100$ based on Illumina MethylationEPIC benchmarking datasets in Settings 1a and 1b. The printed numbers are the mean values of each method.

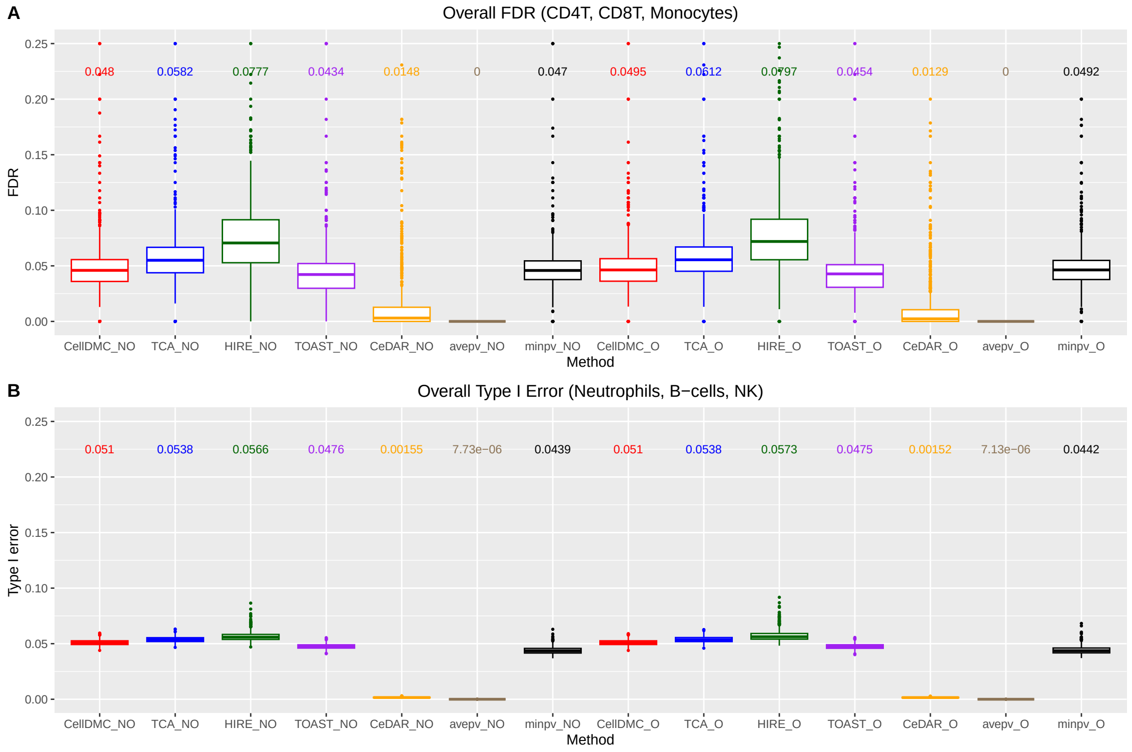

Figure S16: A. Boxplots of empirical FDR across different methods aggregating results for cell-type-specific differential methylation analysis in CD4T, CD8T and monocytes, and SNRs for $n = 100$ based on Illumina MethylationEPIC benchmarking datasets in Settings 2a (NO: no overlapping cell-type-specific effect CpGs) and 2b (O: overlapping cell-type-specific effect CpGs in CD4T and CD8T). B . Boxplots of empirical type I error across different methods aggregating results for cell-type-specific differential methylation analysis in neutrophils, B-cells and NK cells, and SNRs for $n = 100$ based on Illumina MethylationEPIC benchmarking datasets in Settings 2a and 2b. The printed numbers are the mean values of each method.

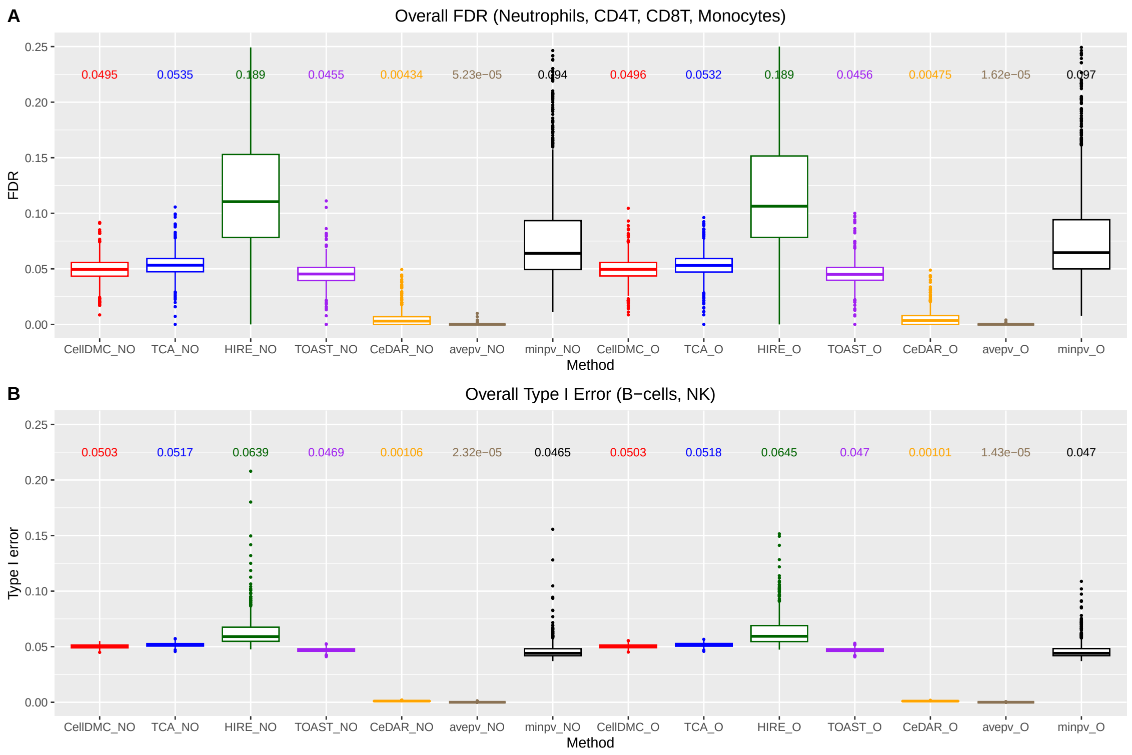

Figure S17: A. Boxplots of empirical FDR across different methods aggregating results for cell-type-specific differential methylation analysis in neutrophils, CD4T, CD8T and monocytes, and SNRs for $n = 200$ based on Illumina MethylationEPIC benchmarking datasets in Settings 1a (NO: no overlapping cell-type-specific effect CpGs) and 1b (O: overlapping cell-type-specific effect CpGs in CD4T and CD8T). B . Boxplots of empirical type I error across different methods aggregating results for cell-type-specific differential methylation analysis in B-cells and NK cells, and SNRs for $n = 200$ based on Illumina MethylationEPIC benchmarking datasets in Settings 1a and 1b. The printed numbers are the mean values of each method.

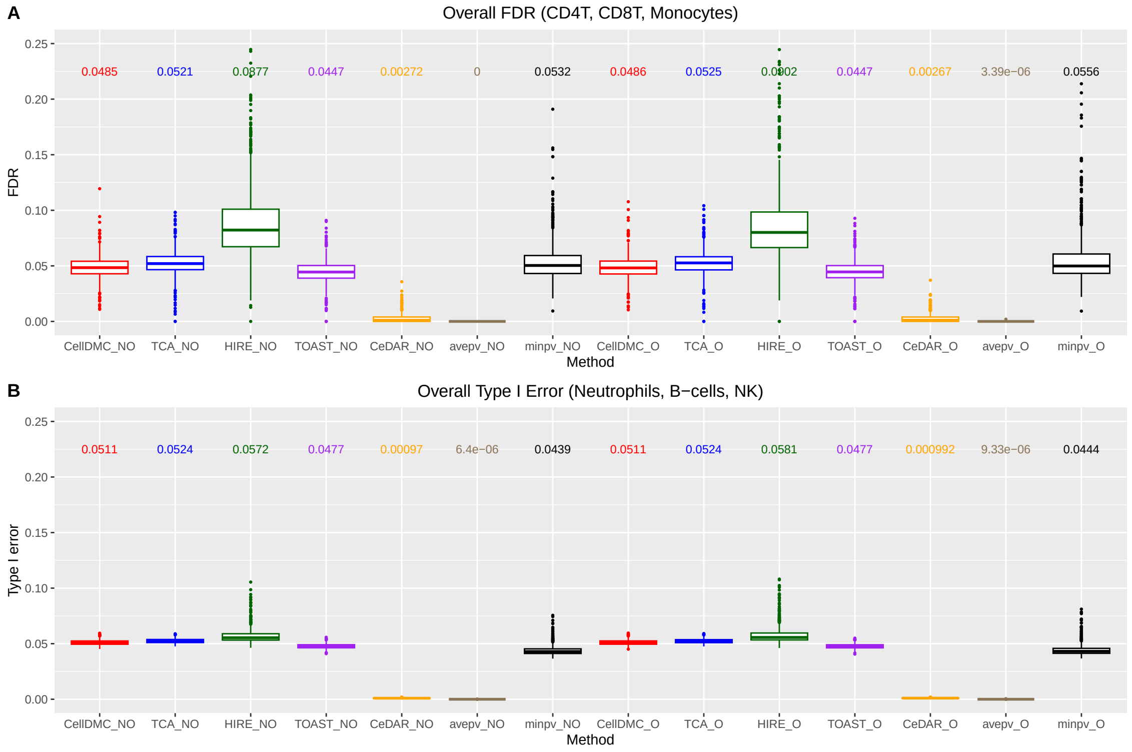

Figure S18: A. Boxplots of empirical FDR across different methods aggregating results for cell-type-specific differential methylation analysis in CD4T, CD8T and monocytes, and SNRs for $n = 200$ based on Illumina MethylationEPIC benchmarking datasets in Settings 2a (NO: no overlapping cell-type-specific effect CpGs) and 2b (O: overlapping cell-type-specific effect CpGs in CD4T and CD8T). B . Boxplots of empirical type I error across different methods aggregating results for cell-type-specific differential methylation analysis in neutrophils, B-cells and NK cells, and SNRs for $n = 200$ based on Illumina MethylationEPIC benchmarking datasets in Settings 2a and 2b. The printed numbers are the mean values of each method.

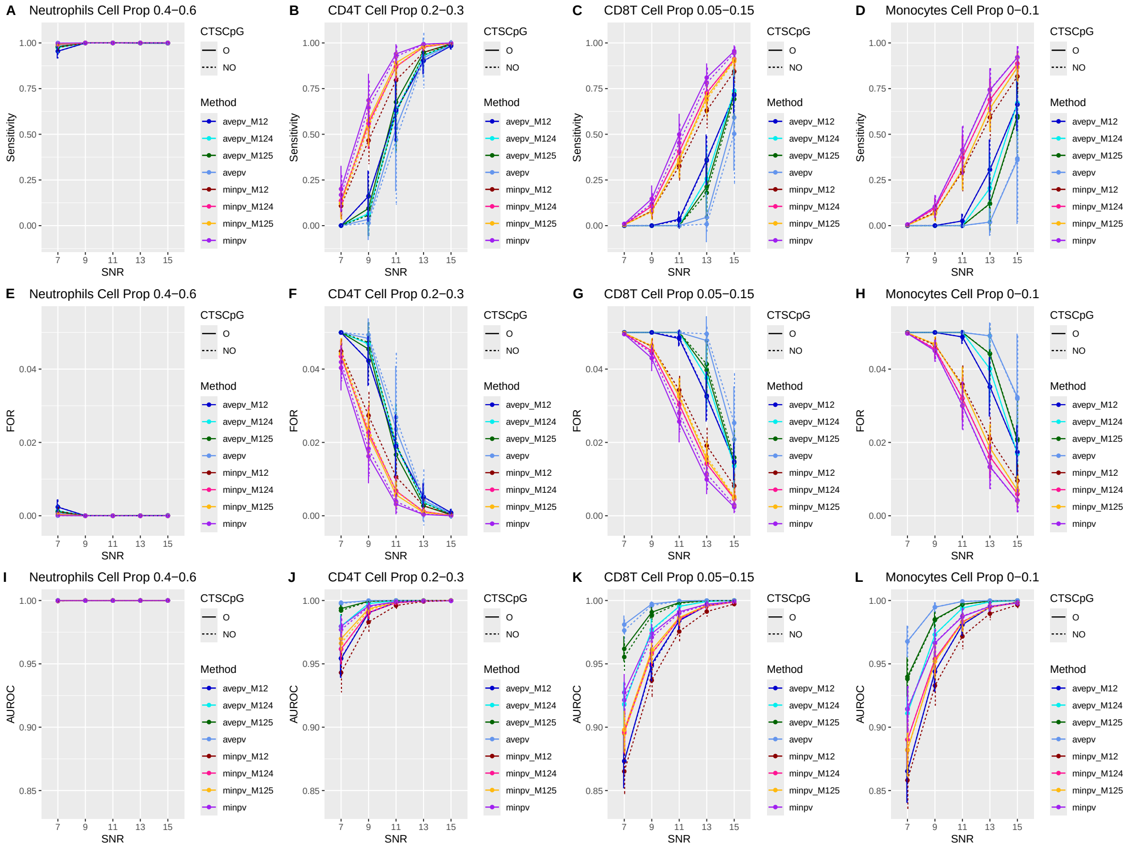

Figure S19: Sensitivity, FOR and AUROC across different result aggregation methods and cell type proportions for $n = 100$ based on Illumina MethylationEPIC benchmarking datasets in Settings 1a (NO: no overlapping cell-type-specific effect CpGs) and 1b (O: overlapping cell-type-specific effect CpGs in CD4T and CD8T) (*minpv* and *avepv* refer to aggregating results from all five methods). A-C. Sensitivity plots for cell-type-specific differential methylation analysis in neutrophils, CD4T, CD8T and monocytes, respectively. D-F. FOR plots. G-I. AUROC plots.

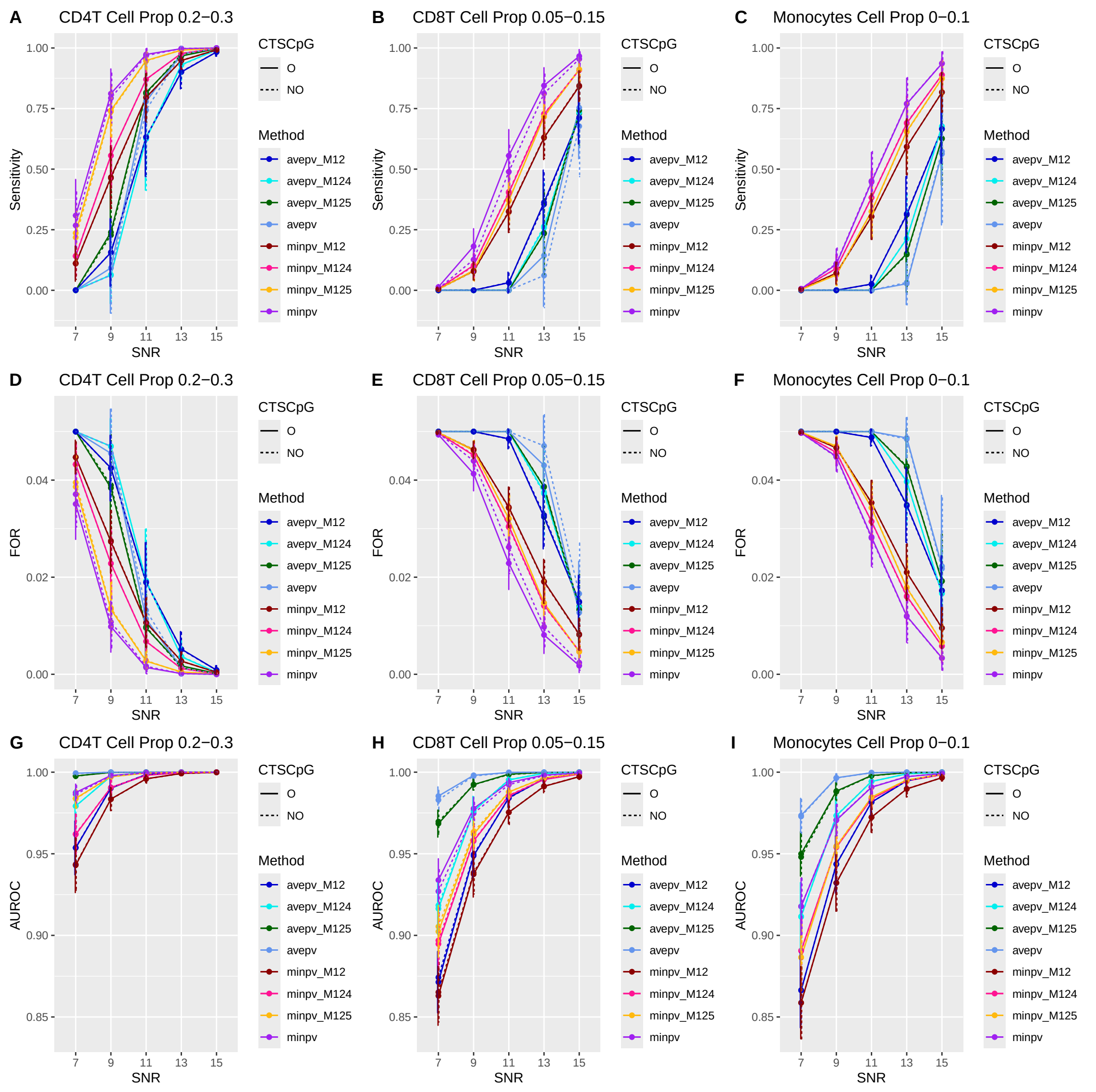

Figure S20: Sensitivity, FOR and AUROC across different result aggregation methods and cell type proportions for $n = 100$ based on Illumina MethylationEPIC benchmarking datasets in Settings 2a (NO: no overlapping cell-type-specific effect CpGs) and 2b (O: overlapping cell-type-specific effect CpGs in CD4T and CD8T) (*minpv* and *avepv* refer to aggregating results from all five methods). A-C. Sensitivity plots for cell-type-specific differential methylation analysis in CD4T, CD8T and monocytes, respectively. D-F. FOR plots. G-I. AUROC plots.

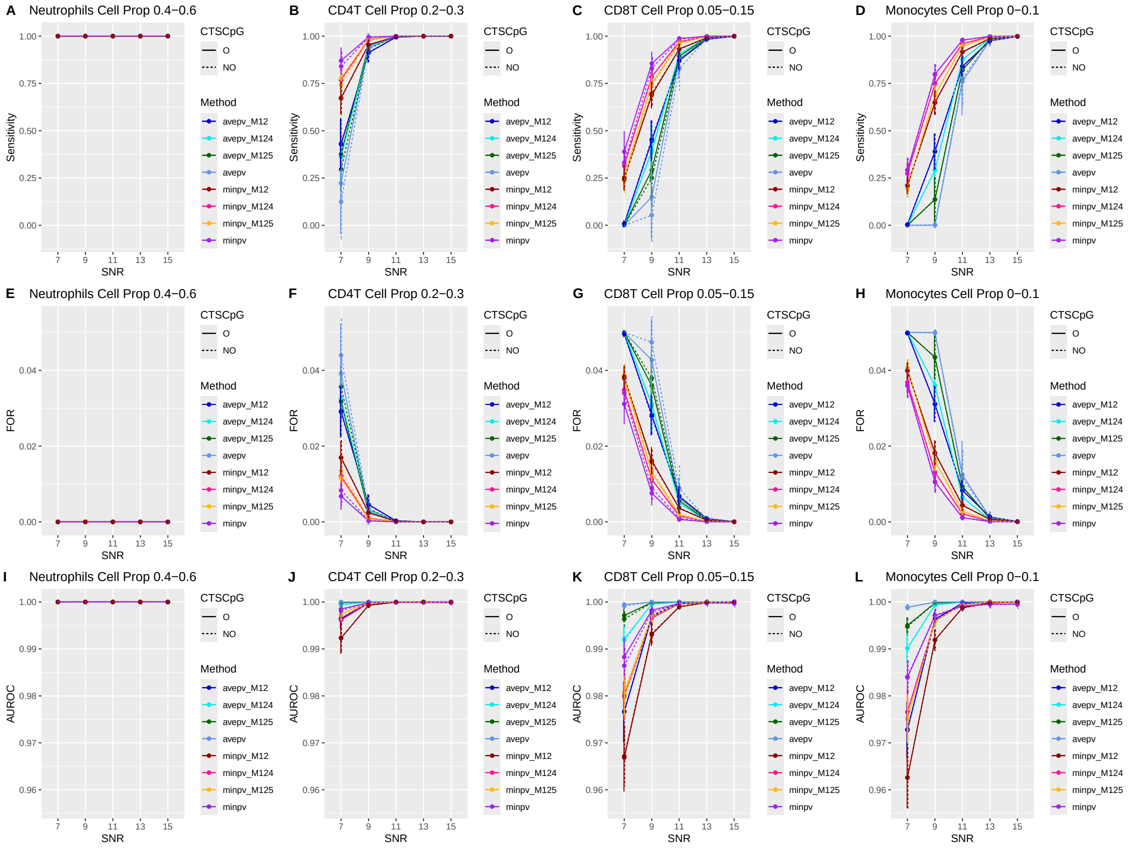

Figure S21: Sensitivity, FOR and AUROC across different result aggregation methods and cell type proportions for $n = 200$ based on Illumina MethylationEPIC benchmarking datasets in Settings 1a (NO: no overlapping cell-type-specific effect CpGs) and 1b (O: overlapping cell-type-specific effect CpGs in CD4T and CD8T) (*minpv* and *avepv* refer to aggregating results from all five methods). A-C. Sensitivity plots for cell-type-specific differential methylation analysis in neutrophils, CD4T, CD8T and monocytes, respectively. D-F. FOR plots. G-I. AUROC plots.

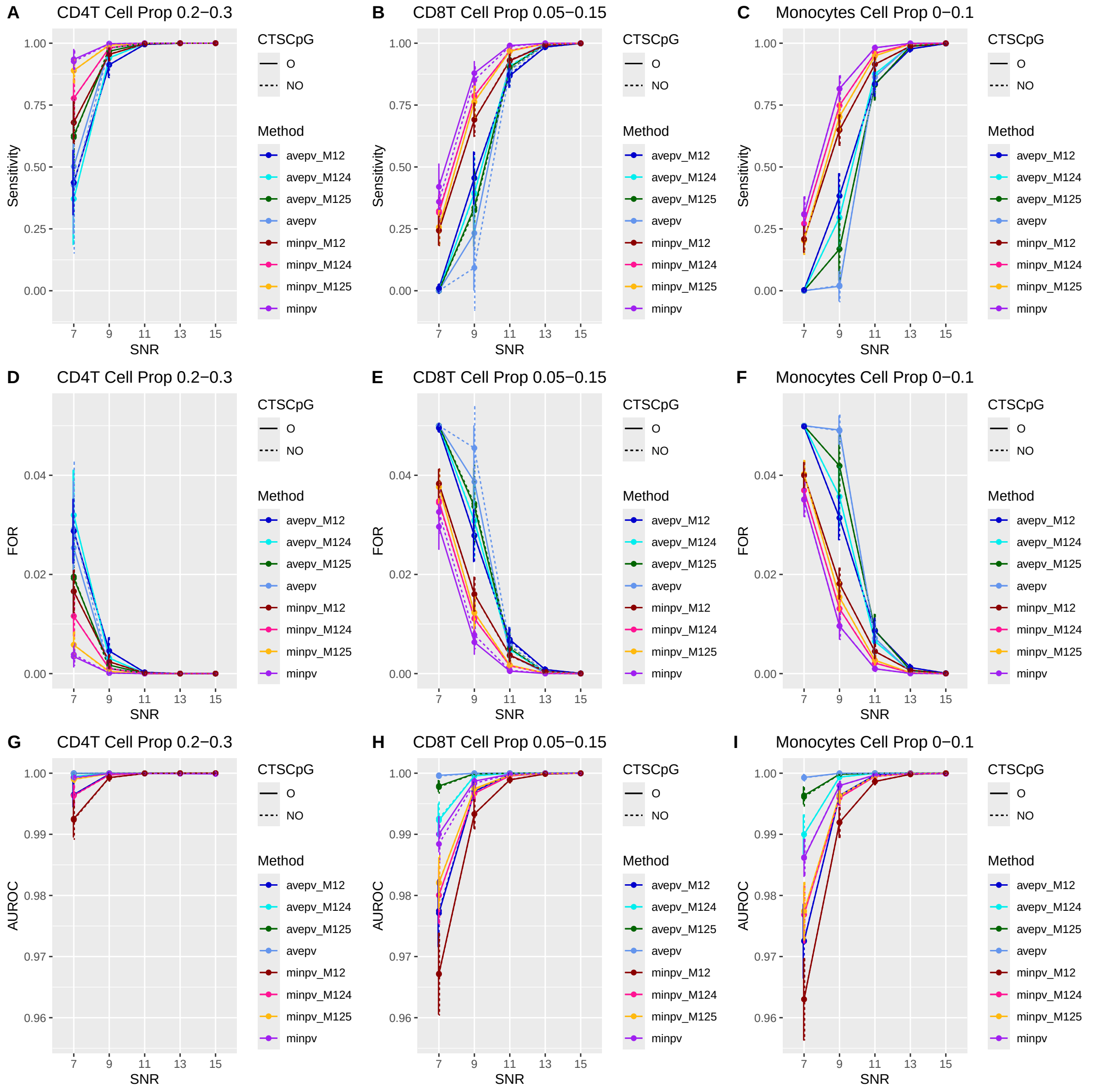

Figure S22: Sensitivity, FOR and AUROC across different result aggregation methods and cell type proportions for $n = 200$ based on Illumina MethylationEPIC benchmarking datasets in Settings 2a (NO: no overlapping cell-type-specific effect CpGs) and 2b (O: overlapping cell-type-specific effect CpGs in CD4T and CD8T) (*minpv* and *avepv* refer to aggregating results from all five methods). A-C. Sensitivity plots for cell-type-specific differential methylation analysis in CD4T, CD8T and monocytes, respectively. D-F. FOR plots. G-I. AUROC plots.

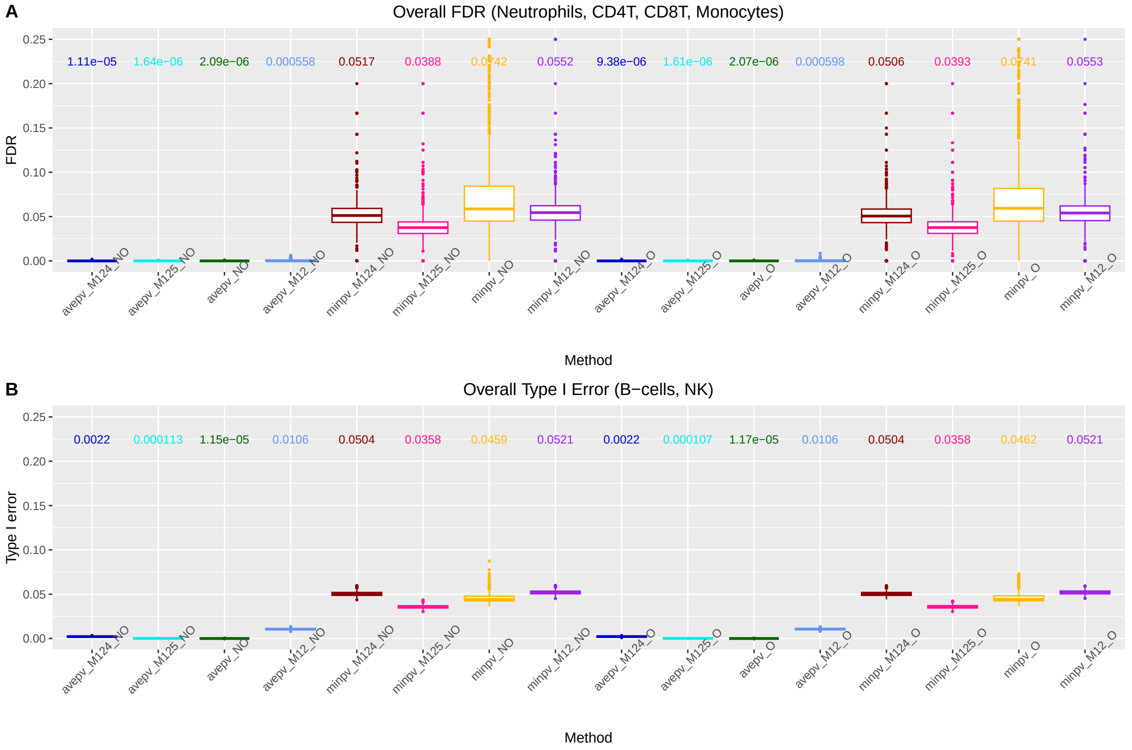

Figure S23: A. Boxplots of empirical FDR across different result aggregation methods, aggregating results for cell-type-specific differential methylation analysis in neutrophils, CD4T, CD8T and monocytes, and SNRs for $n = 100$ based on Illumina MethylationEPIC benchmarking datasets in Settings 1a (NO: no overlapping cell-type-specific effect CpGs) and 1b (O: overlapping cell-type-specific effect CpGs in CD4T and CD8T) (*minpv* and *avepv* refer to aggregating results from all five methods). B . Boxplots of empirical type I error across different result aggregation methods, aggregating results for cell-type-specific differential methylation analysis in B-cells and NK cells, and SNRs for $n = 100$ based on Illumina MethylationEPIC benchmarking datasets in Settings 1a and 1b. The printed numbers are the mean values of each method.

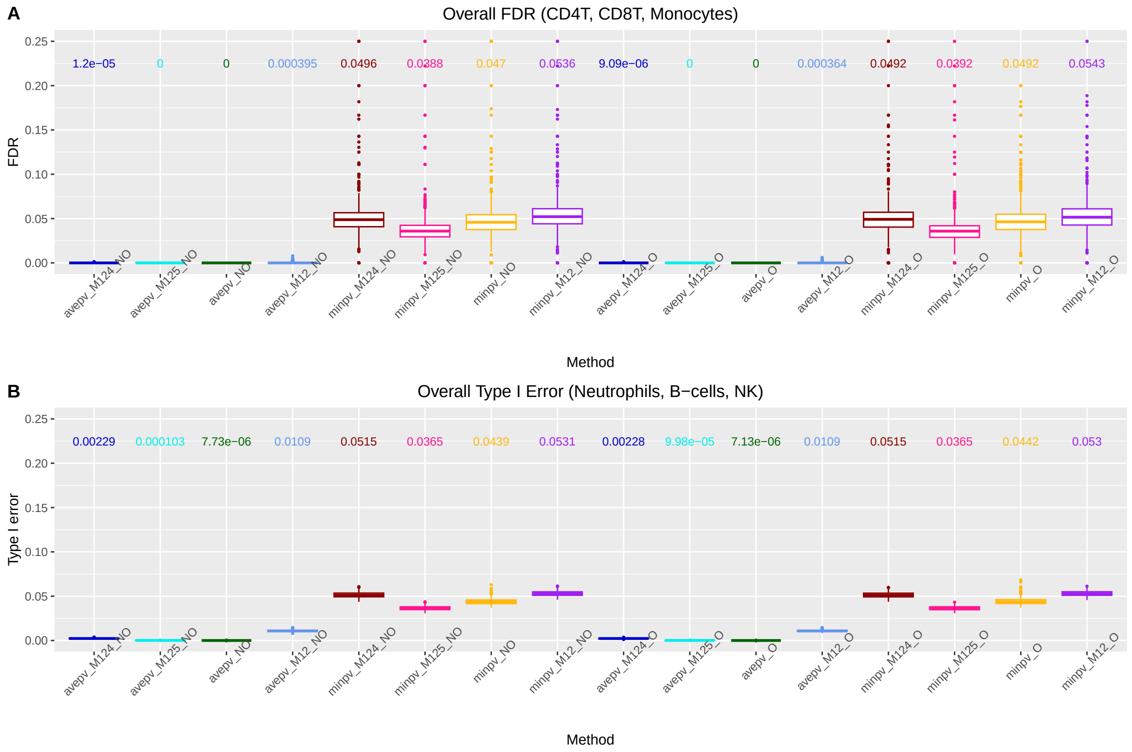

Figure S24: A. Boxplots of empirical FDR across different result aggregation methods, aggregating results for cell-type-specific differential methylation analysis in CD4T, CD8T and monocytes, and SNRs for $n = 100$ based on Illumina MethylationEPIC benchmarking datasets in Settings 2a (NO: no overlapping cell-type-specific effect CpGs) and 2b (O: overlapping cell-type-specific effect CpGs in CD4T and CD8T) (*minpv* and *avepv* refer to aggregating results from all five methods). B . Boxplots of empirical type I error across different result aggregation methods, aggregating results for cell-type-specific differential methylation analysis in neutrophils, B-cells and NK cells, and SNRs for $n = 100$ based on Illumina MethylationEPIC benchmarking datasets in Settings 2a and 2b. The printed numbers are the mean values of each method.

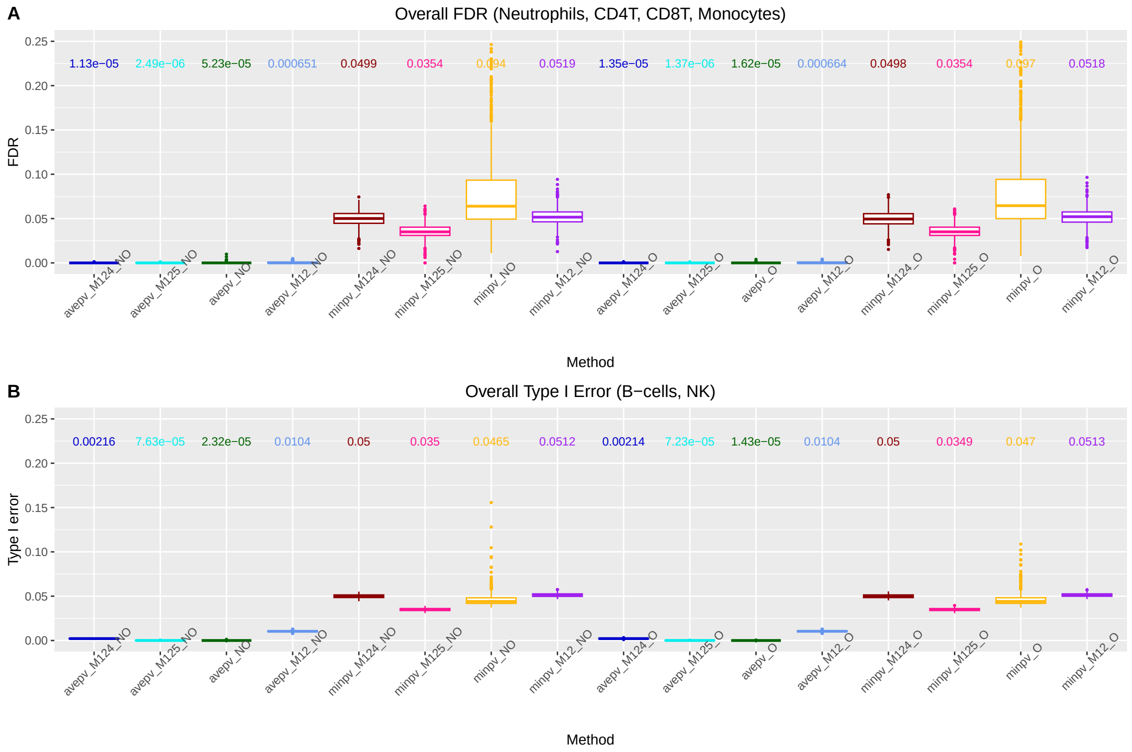

Figure S25: A. Boxplots of empirical FDR across different result aggregation methods, aggregating results for cell-type-specific differential methylation analysis in neutrophils, CD4T, CD8T and monocytes, and SNRs for $n = 200$ based on Illumina MethylationEPIC benchmarking datasets in Settings 1a (NO: no overlapping cell-type-specific effect CpGs) and 1b (O: overlapping cell-type-specific effect CpGs in CD4T and CD8T) (*minpv* and *avepv* refer to aggregating results from all five methods). B . Boxplots of empirical type I error across different result aggregation methods, aggregating results for cell-type-specific differential methylation analysis in B-cells and NK cells, and SNRs for $n = 200$ based on Illumina MethylationEPIC benchmarking datasets in Settings 1a and 1b. The printed numbers are the mean values of each method.

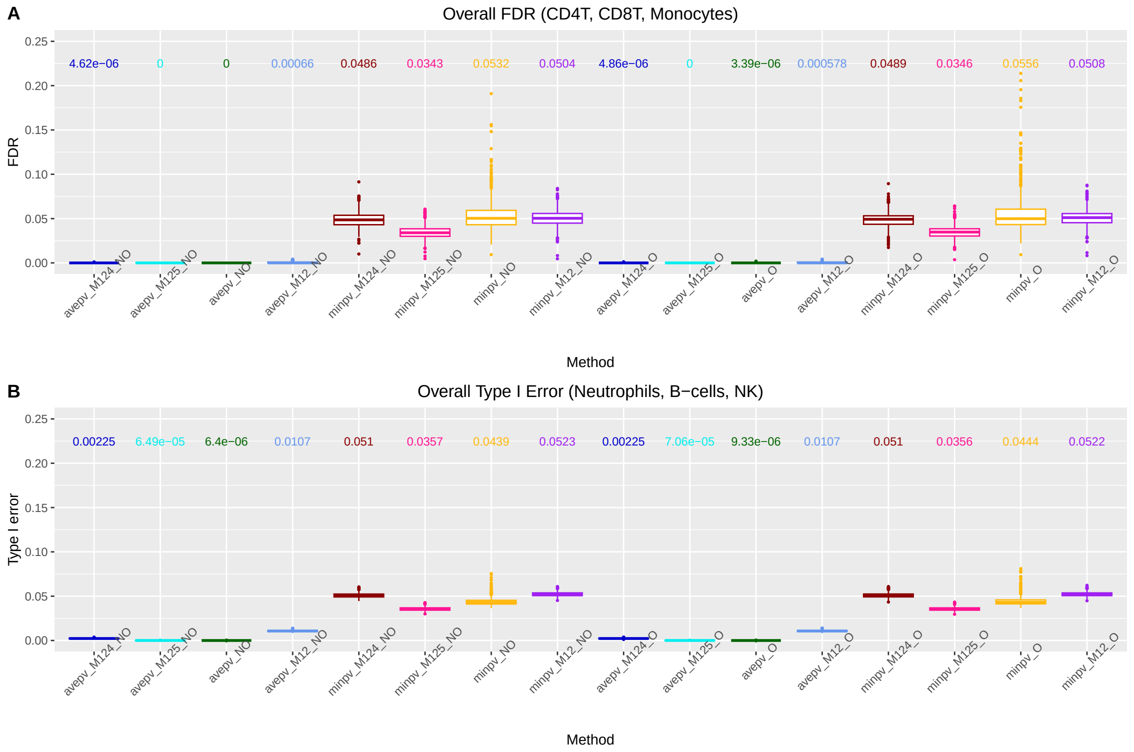

Figure S26: A. Boxplots of empirical FDR across different result aggregation methods, aggregating results for cell-type-specific differential methylation analysis in CD4T, CD8T and monocytes, and SNRs for $n = 200$ based on Illumina MethylationEPIC benchmarking datasets in Settings 2a (NO: no overlapping cell-type-specific effect CpGs) and 2b (O: overlapping cell-type-specific effect CpGs in CD4T and CD8T) (*minpv* and *avepv* refer to aggregating results from all five methods). B . Boxplots of empirical type I error across different result aggregation methods, aggregating results for cell-type-specific differential methylation analysis in neutrophils, B-cells and NK cells, and SNRs for $n = 200$ based on Illumina MethylationEPIC benchmarking datasets in Settings 2a and 2b. The printed numbers are the mean values of each method.

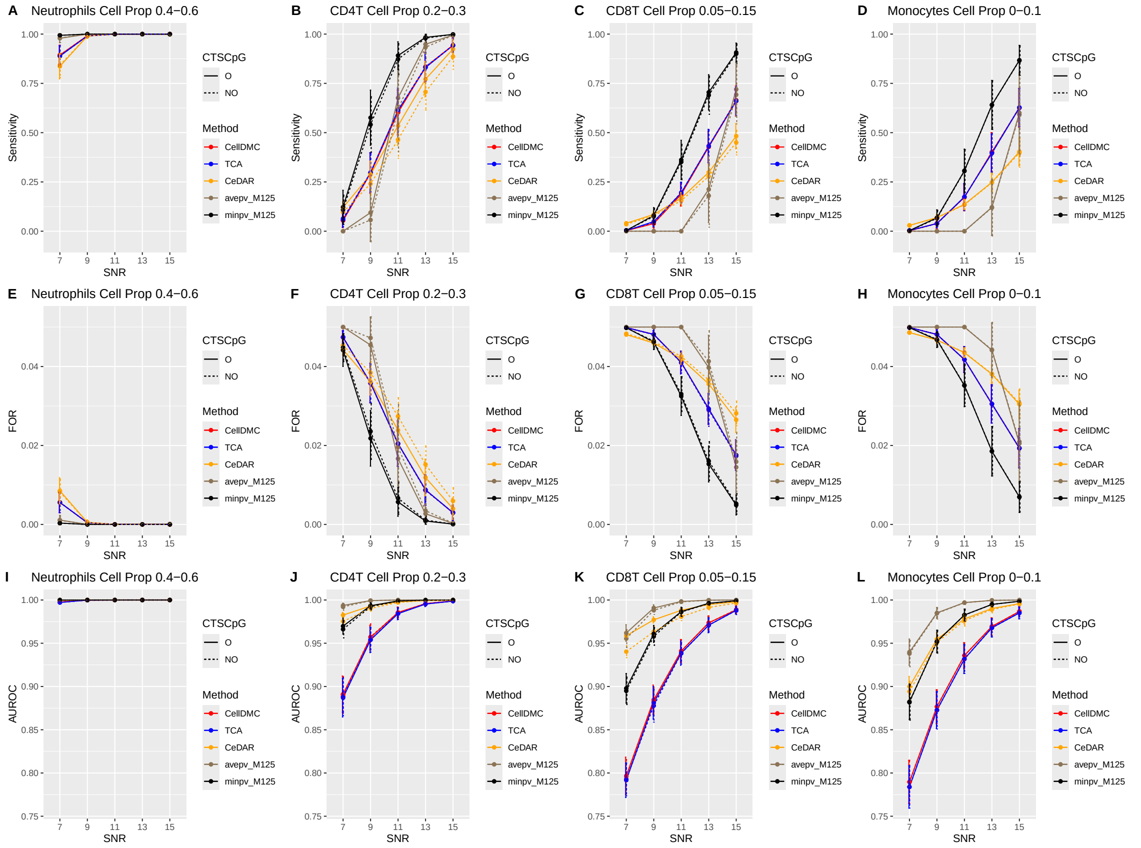

Figure S27: Sensitivity, FOR and AUROC across different result aggregation methods and cell type proportions for $n = 100$ comparing CellDMC, TCA, CeDAR and result aggregation methods which combine these three methods based on Illumina MethylationEPIC benchmarking datasets in Settings 1a (NO: no overlapping cell-type-specific effect CpGs) and 1b (O: overlapping cell-type-specific effect CpGs in CD4T and CD8T). A-C. Sensitivity plots for cell-type-specific differential methylation analysis in neutrophils, CD4T, CD8T and monocytes, respectively. D-F. FOR plots. G-I. AUROC plots.

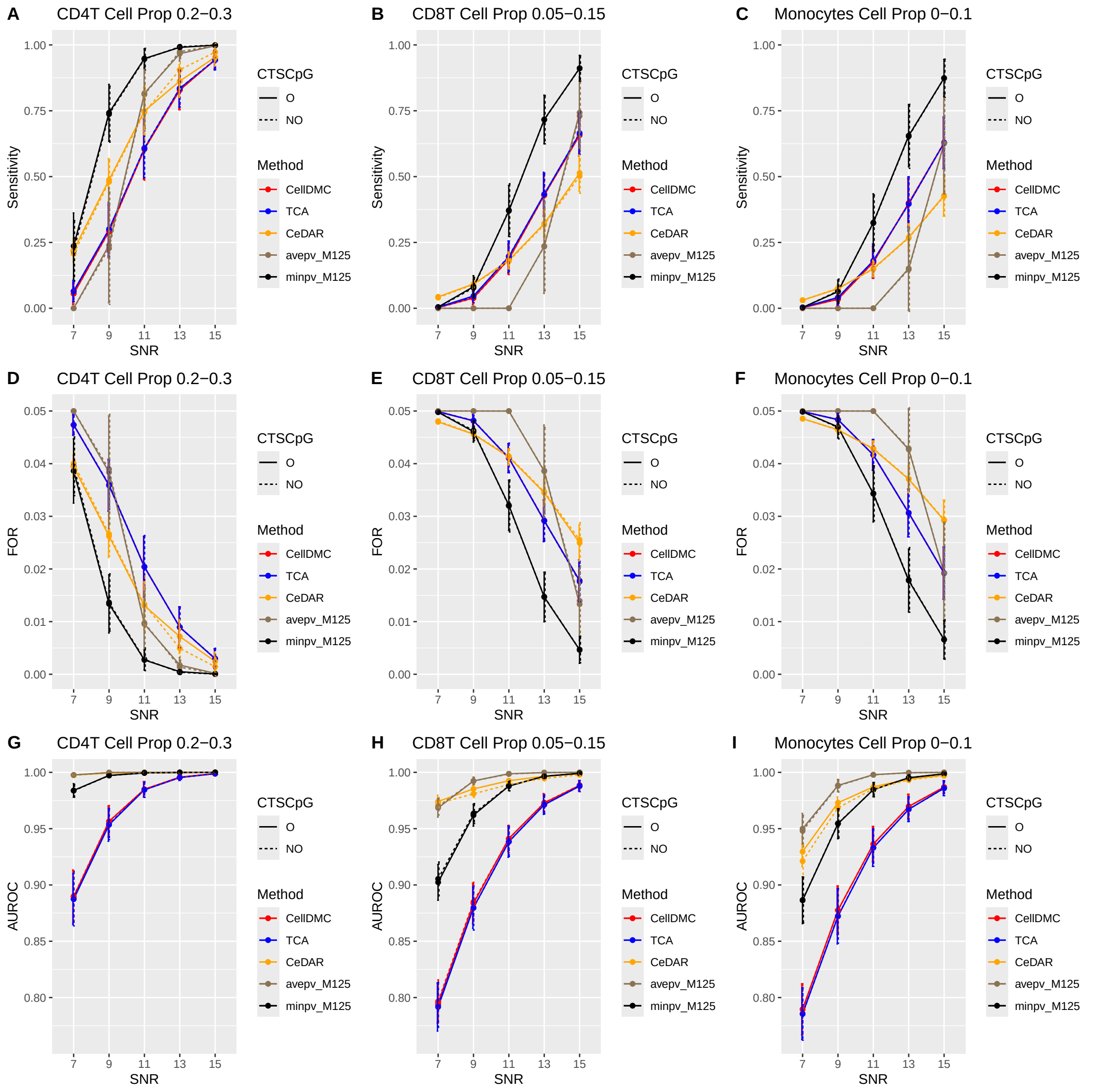

Figure S28: Sensitivity, FOR and AUROC across different result aggregation methods and cell type proportions for $n = 100$ comparing CellDMC, TCA, CeDAR and result aggregation methods which combine these three methods based on Illumina MethylationEPIC benchmarking datasets in Settings 2a (NO: no overlapping cell-type-specific effect CpGs) and 2b (O: overlapping cell-type-specific effect CpGs in CD4T and CD8T). A-C. Sensitivity plots for cell-type-specific differential methylation analysis in CD4T, CD8T and monocytes, respectively. D-F. FOR plots. G-I. AUROC plots.

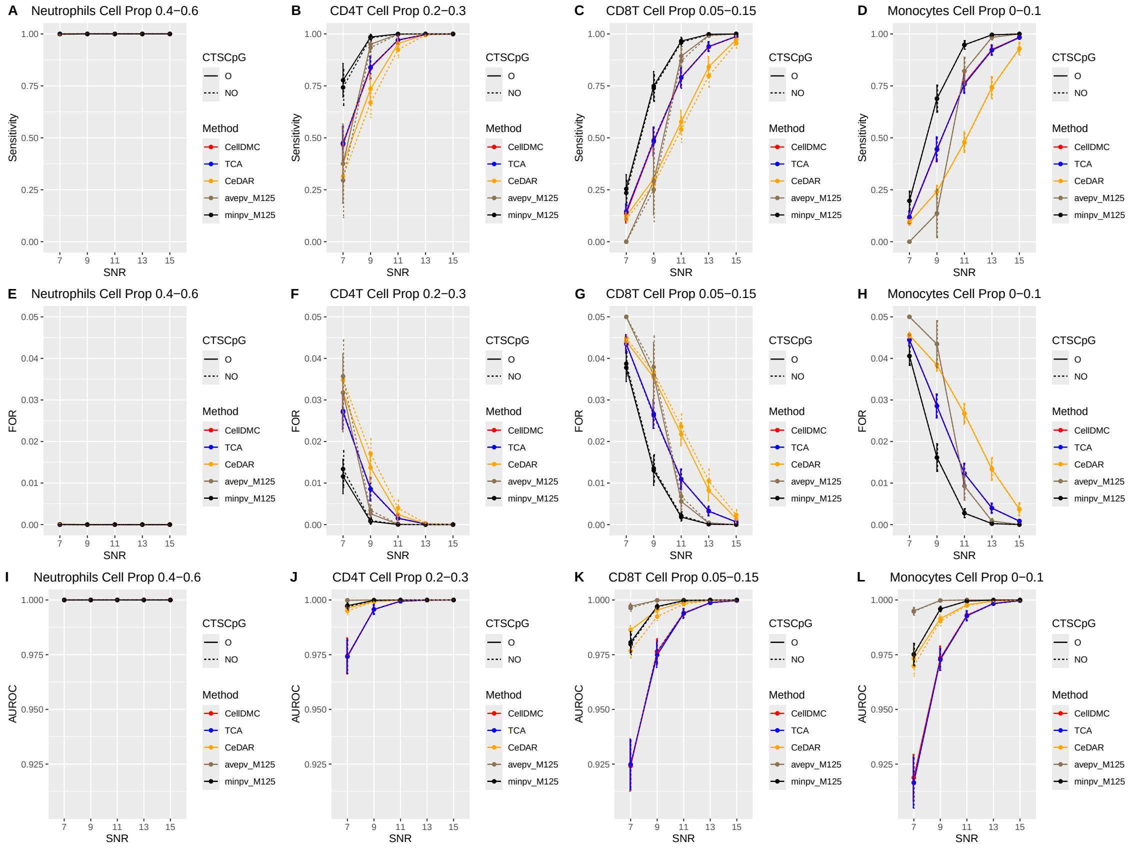

Figure S29: Sensitivity, FOR and AUROC across different result aggregation methods and cell type proportions for $n = 200$ comparing CellDMC, TCA, CeDAR and result aggregation methods which combine these three methods based on Illumina MethylationEPIC benchmarking datasets in Settings 1a (NO: no overlapping cell-type-specific effect CpGs) and 1b (O: overlapping cell-type-specific effect CpGs in CD4T and CD8T). A-C. Sensitivity plots for cell-type-specific differential methylation analysis in neutrophils, CD4T, CD8T and monocytes, respectively. D-F. FOR plots. G-I. AUROC plots.

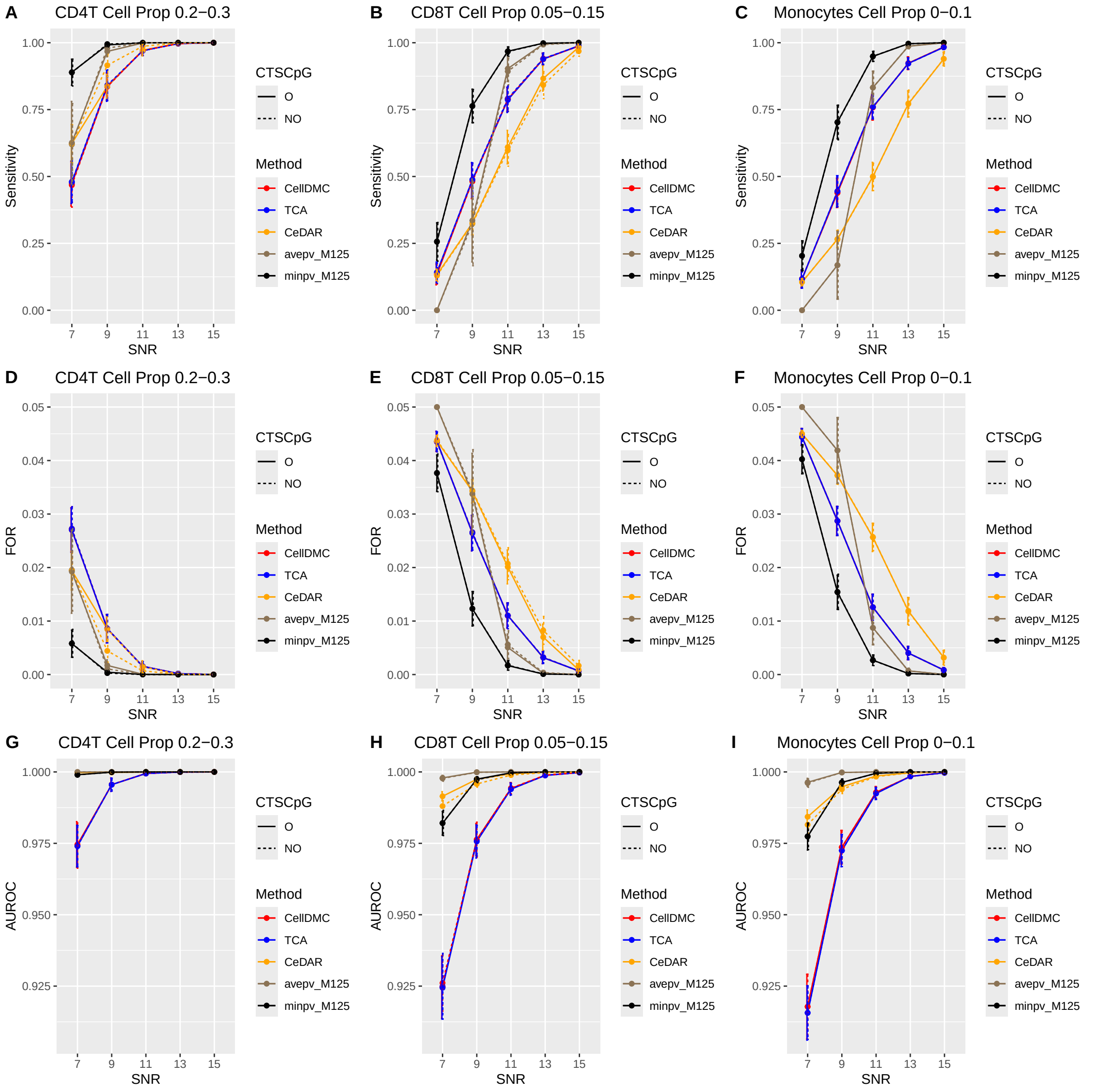

Figure S30: Sensitivity, FOR and AUROC across different result aggregation methods and cell type proportions for $n = 200$ comparing CellDMC, TCA, CeDAR and result aggregation methods which combine these three methods based on Illumina MethylationEPIC benchmarking datasets in Settings 2a (NO: no overlapping cell-type-specific effect CpGs) and 2b (O: overlapping cell-type-specific effect CpGs in CD4T and CD8T). A-C. Sensitivity plots for cell-type-specific differential methylation analysis in CD4T, CD8T and monocytes, respectively. D-F. FOR plots. G-I. AUROC plots.

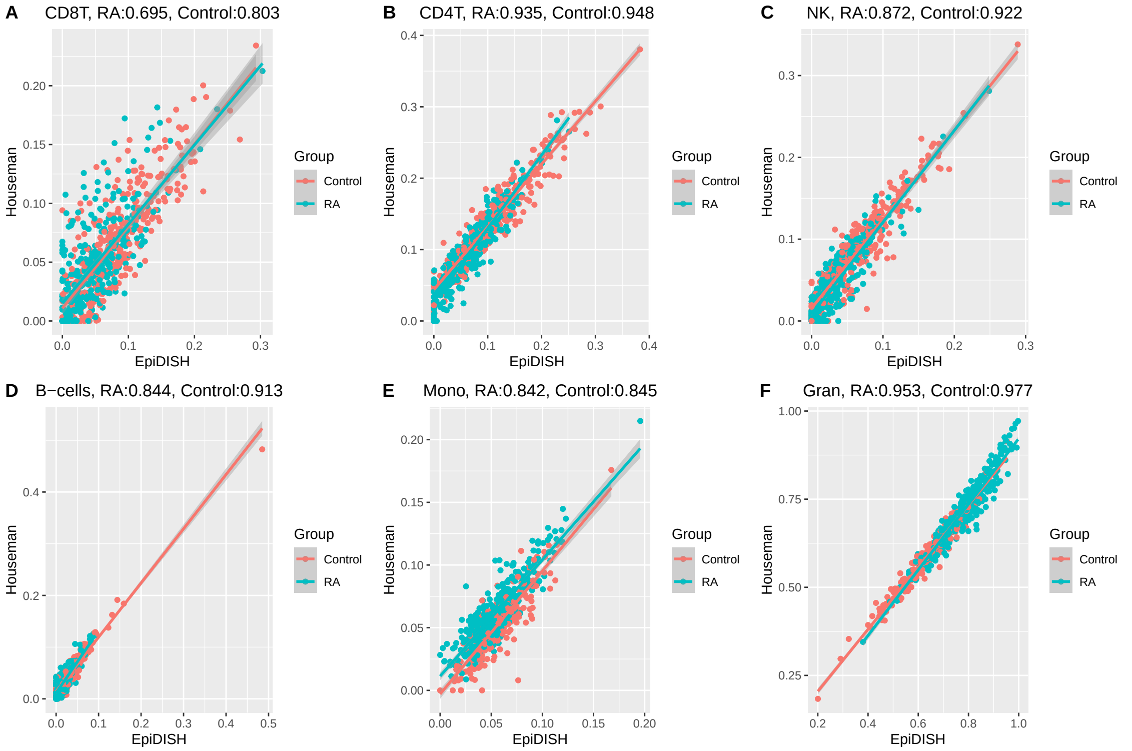

Figure S31: Scatter plots comparing the estimate cell proportions obtained from EpiDISH versus Houseman's method in rheumatoid arthritis case study. Spearman rank correlation coefficients are provided in the header.

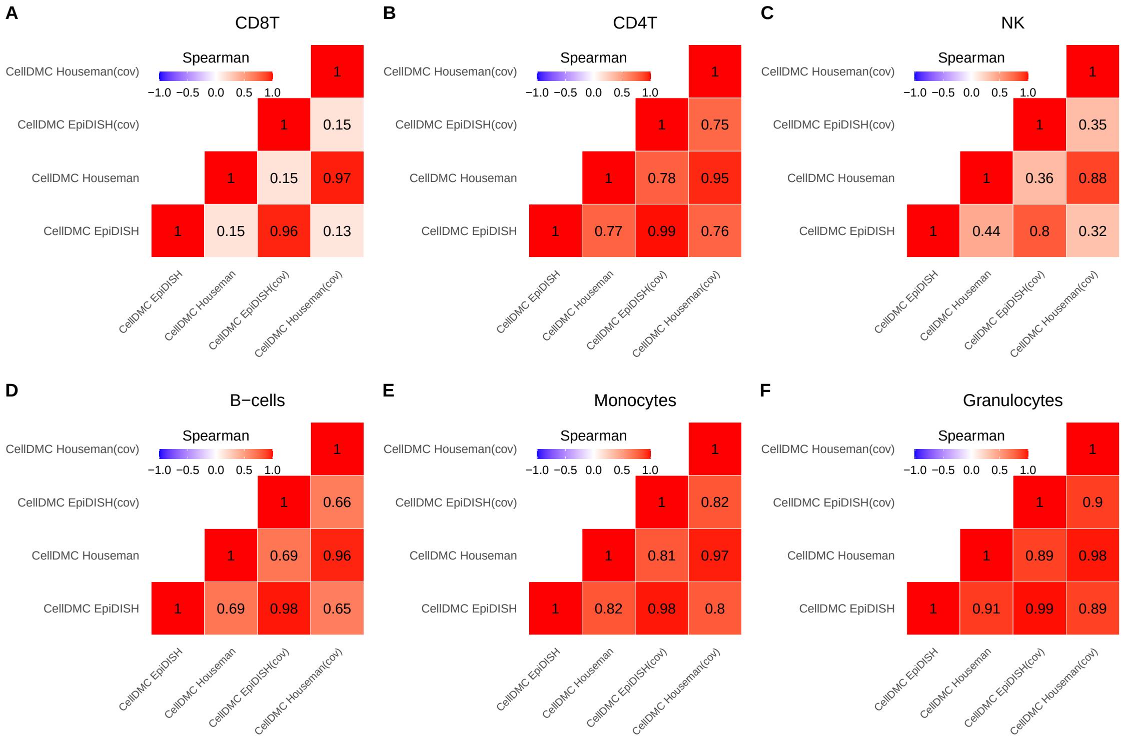

Figure S32: Correlation heatmap comparing the four models using CellDMC within each cell type in rheumatoid arthritis case study, (a) EpiDISH cell proportions without covariate (age, sex and smoking status) adjustment (EpiDISH), (b) Houseman's cell proportions without covariate adjustment (Houseman), (c) EpiDISH cell proportions with covariate adjustment (EpiDISH(cov)), (d) Houseman's cell proportions with covariate adjustment (Houseman(cov)). Spearman rank correlation coefficients are provided in each cell.

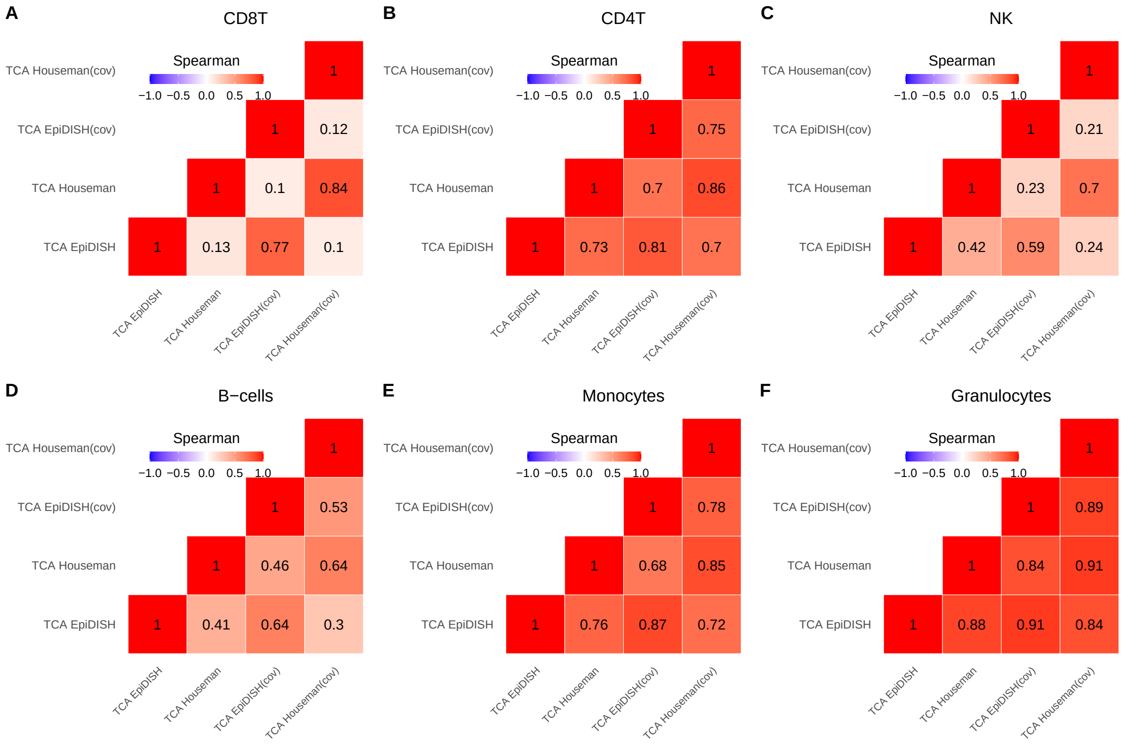

Figure S33: Correlation heatmap comparing the four models using TCA within each cell type in rheumatoid arthritis case study, (a) EpiDISH cell proportions without covariate (age, sex and smoking status) adjustment (EpiDISH), (b) Houseman's cell proportions without covariate adjustment (Houseman), (c) EpiDISH cell proportions with covariate adjustment (EpiDISH(cov)), (d) Houseman's cell proportions with covariate adjustment (Houseman(cov)). Spearman rank correlation coefficients are provided in each cell.

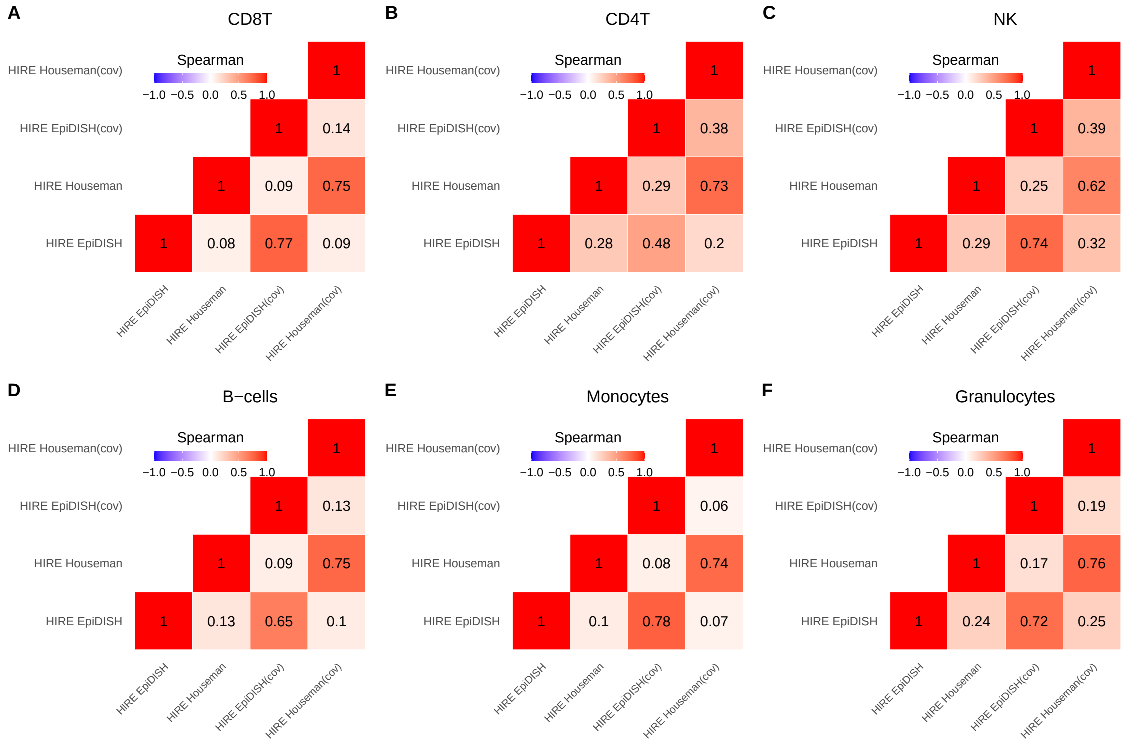

Figure S34: Correlation heatmap comparing the four models using HIRE within each cell type in rheumatoid arthritis case study, (a) EpiDISH cell proportions without covariate (age, sex and smoking status) adjustment (EpiDISH), (b) Houseman's cell proportions without covariate adjustment (Houseman), (c) EpiDISH cell proportions with covariate adjustment (EpiDISH(cov)), (d) Houseman's cell proportions with covariate adjustment (Houseman(cov)). Spearman rank correlation coefficients are provided in each cell.

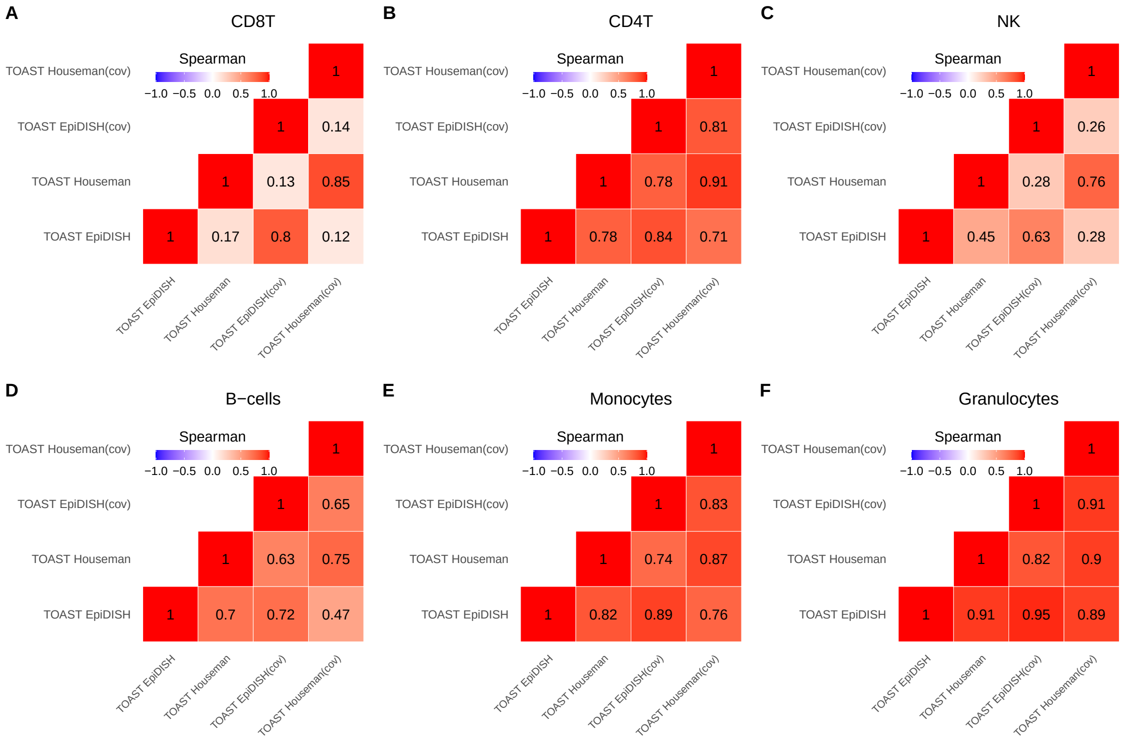

Figure S35: Correlation heatmap comparing the four models using TOAST within each cell type in rheumatoid arthritis case study, (a) EpiDISH cell proportions without covariate (age, sex and smoking status) adjustment (EpiDISH), (b) Houseman's cell proportions without covariate adjustment (Houseman), (c) EpiDISH cell proportions with covariate adjustment (EpiDISH(cov)), (d) Houseman's cell proportions with covariate adjustment (Houseman(cov)). Spearman rank correlation coefficients are provided in each cell.

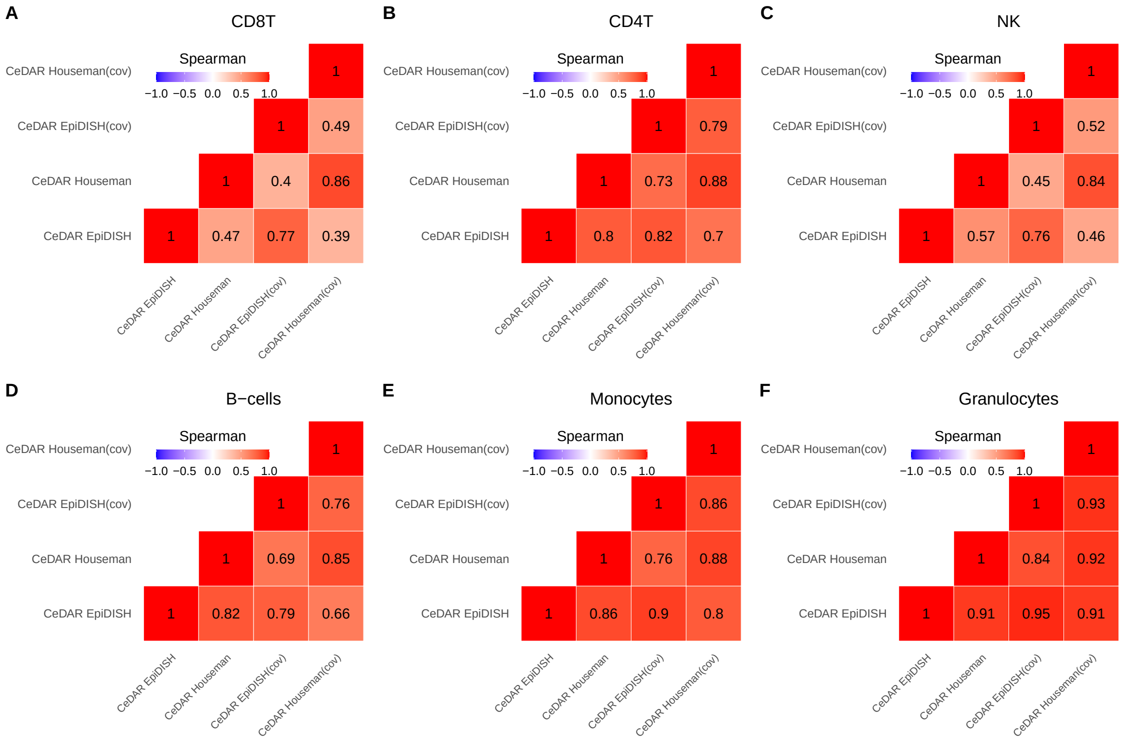

Figure S36: Correlation heatmap comparing the four models using CeDAR within each cell type in rheumatoid arthritis case study, (a) EpiDISH cell proportions without covariate (age, sex and smoking status) adjustment (EpiDISH), (b) Houseman's cell proportions without covariate adjustment (Houseman), (c) EpiDISH cell proportions with covariate adjustment (EpiDISH(cov)), (d) Houseman's cell proportions with covariate adjustment (Houseman(cov)). Spearman rank correlation coefficients are provided in each cell.

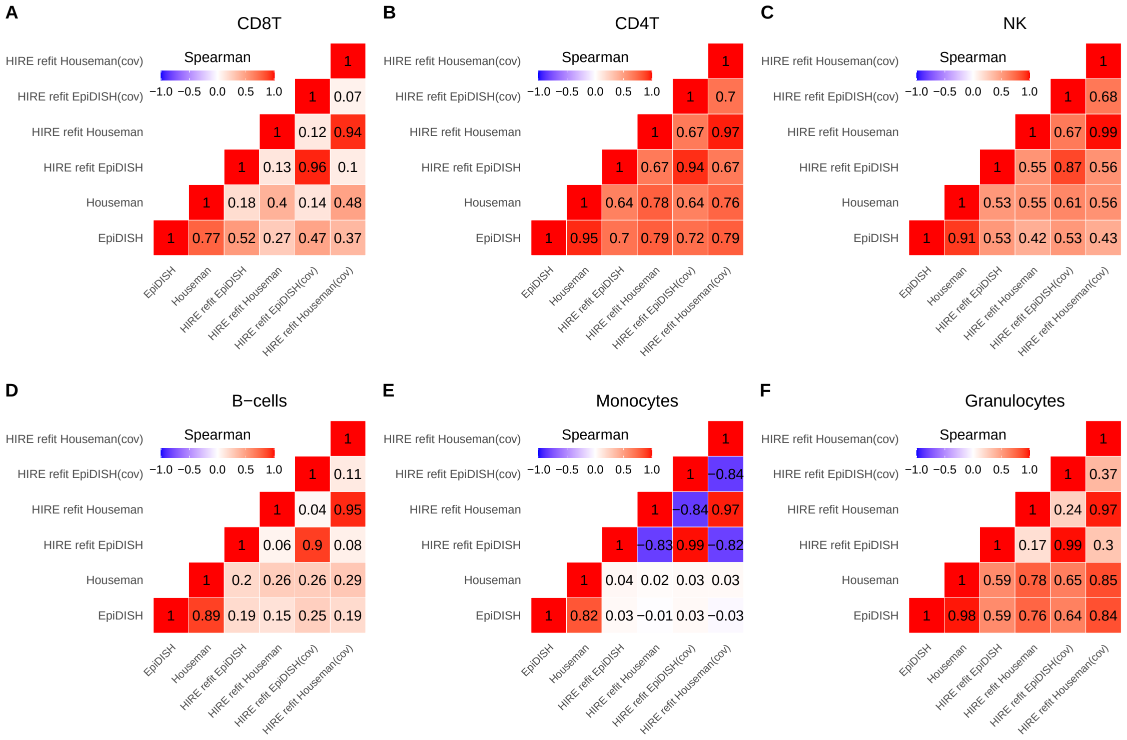

Figure S37: Correlation heatmap comparing the re-estimated cell type proportion in HIRE within each cell type in rheumatoid arthritis case study. Spearman rank correlation coefficients are provided in each cell.

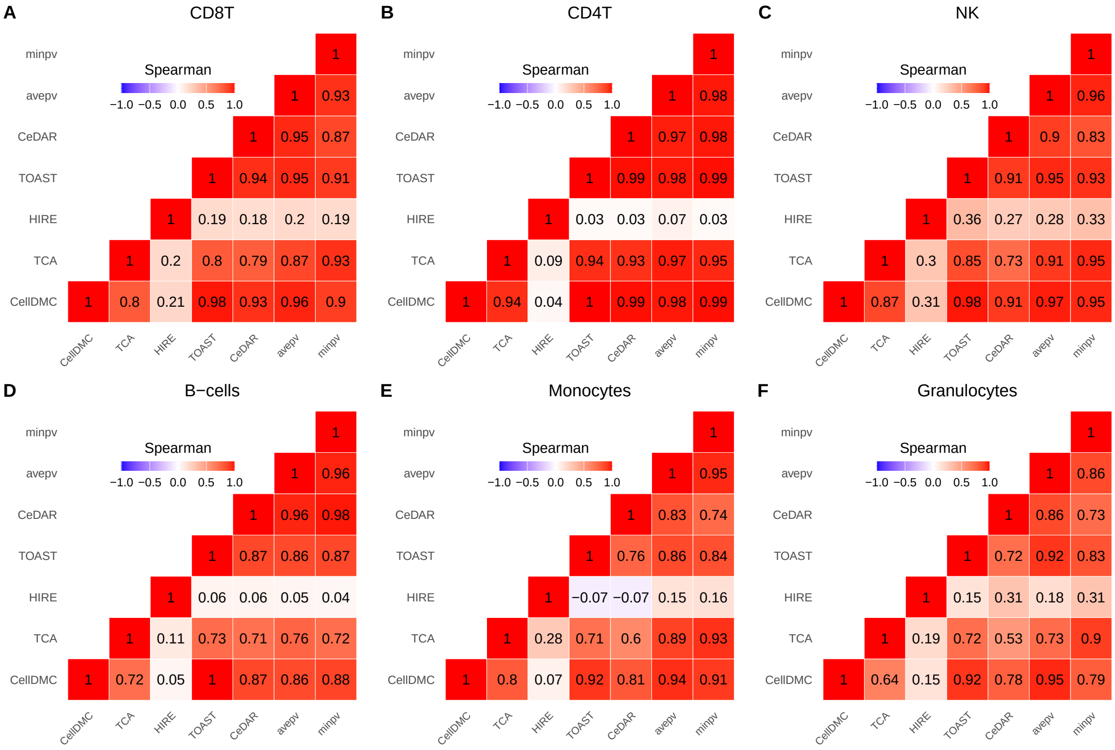

Figure S38: Correlation heatmap comparing the five methods within each cell type in KEGG pathway analysis of rheumatoid arthritis case study. Spearman rank correlation coefficients are provided in each cell.

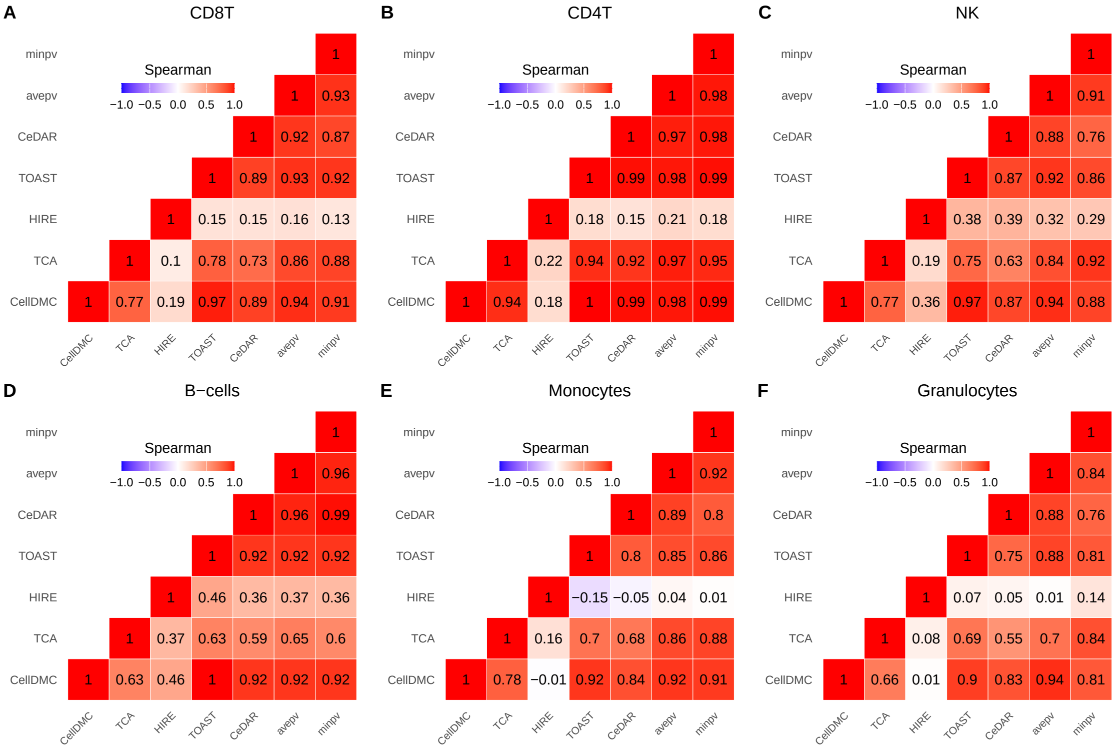

Figure S39: Correlation heatmap comparing the five methods within each cell type in GO pathway analysis of rheumatoid arthritis case study. Spearman rank correlation coefficients are provided in each cell.

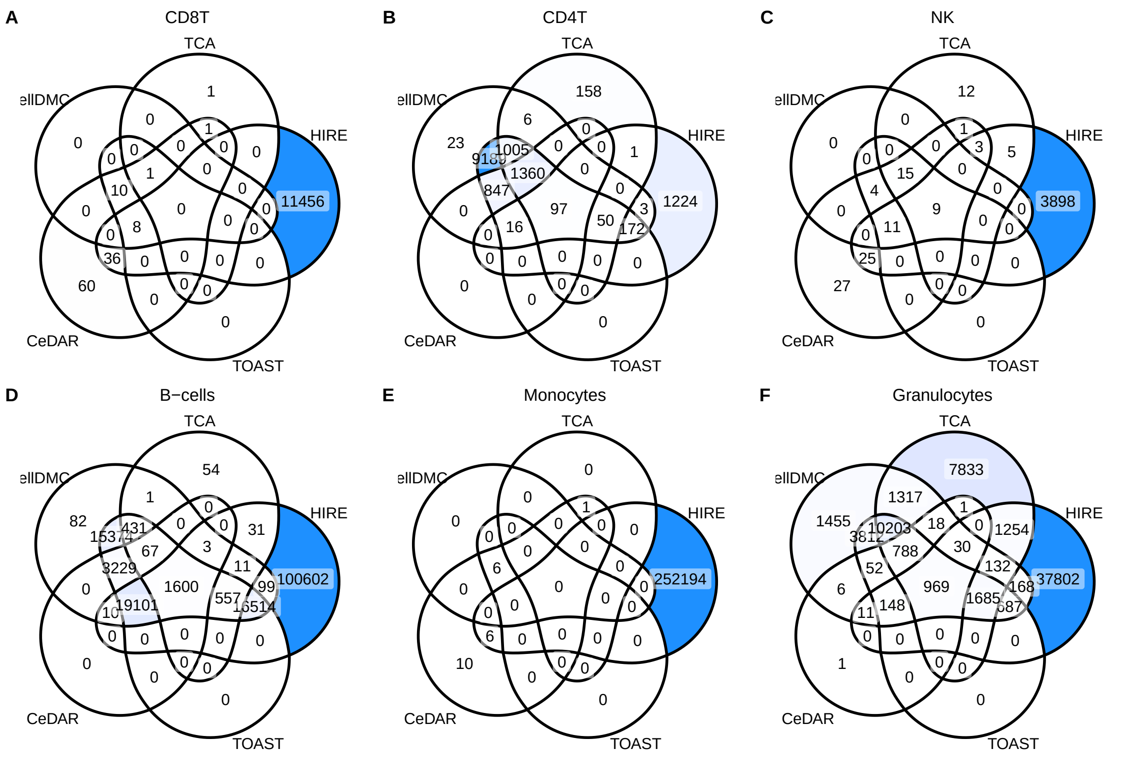

Figure S40: Venn diagram comparing the overlap among the five methods within each cell type in rheumatoid arthritis case study.

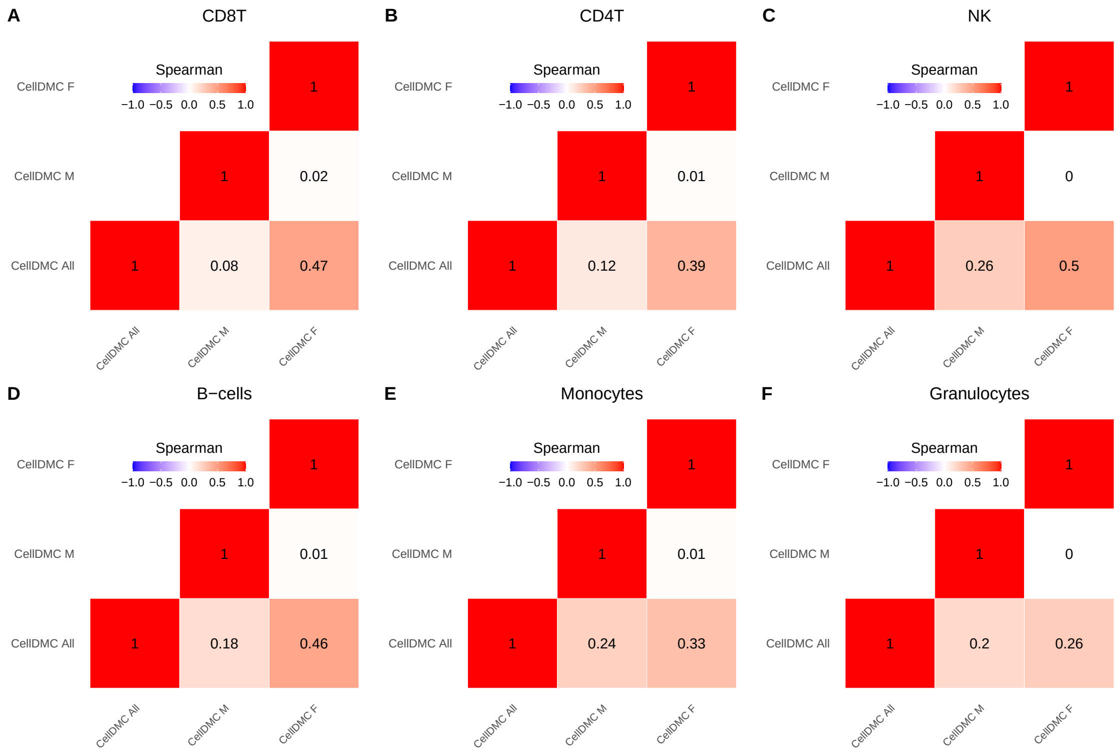

Figure S41: Correlation heatmap comparing the three subsets in CellDMC within each cell type in MDD case study. Spearman rank correlation coefficients are provided in each cell.

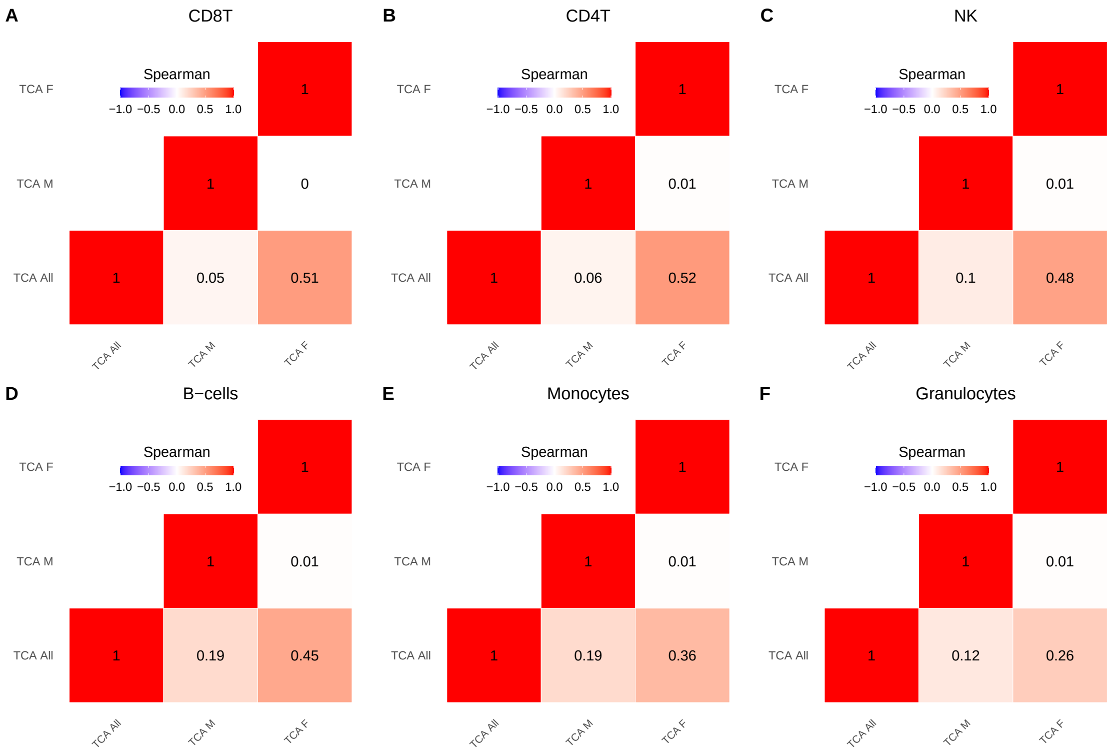

Figure S42: Correlation heatmap comparing the three subsets in TCA within each cell type in MDD case study. Spearman rank correlation coefficients are provided in each cell.

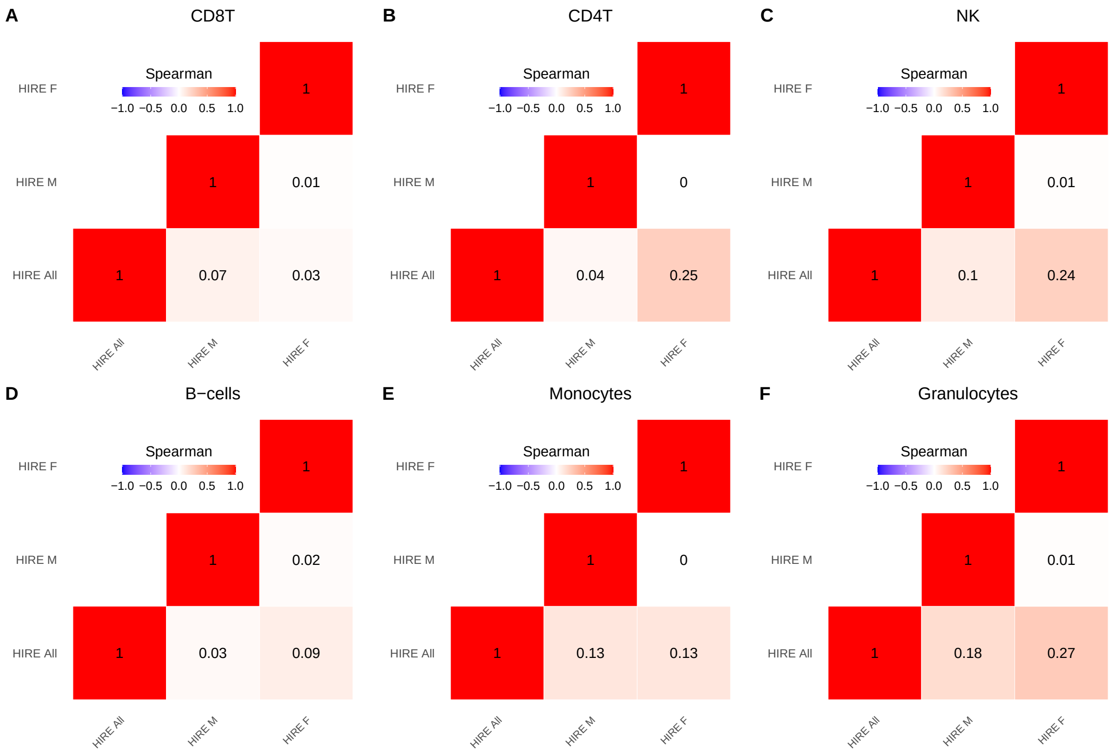

Figure S43: Correlation heatmap comparing the three subsets in HIRE within each cell type in MDD case study. Spearman rank correlation coefficients are provided in each cell.

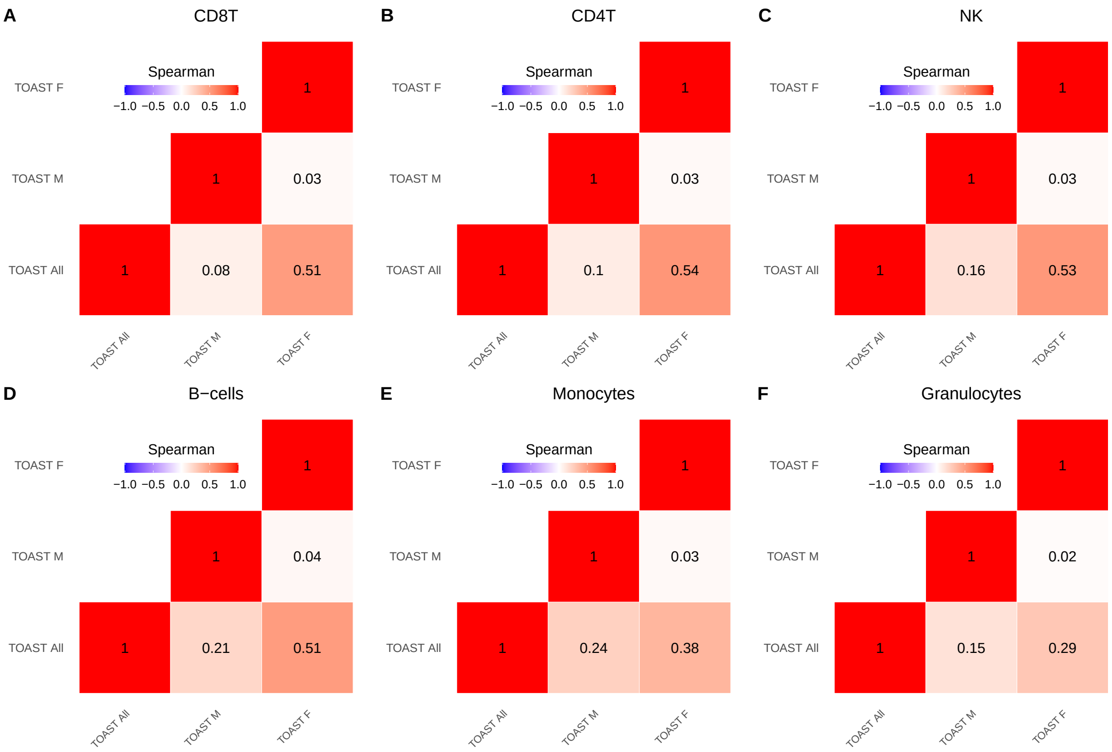

Figure S44: Correlation heatmap comparing the three subsets in TOAST within each cell type in MDD case study. Spearman rank correlation coefficients are provided in each cell.

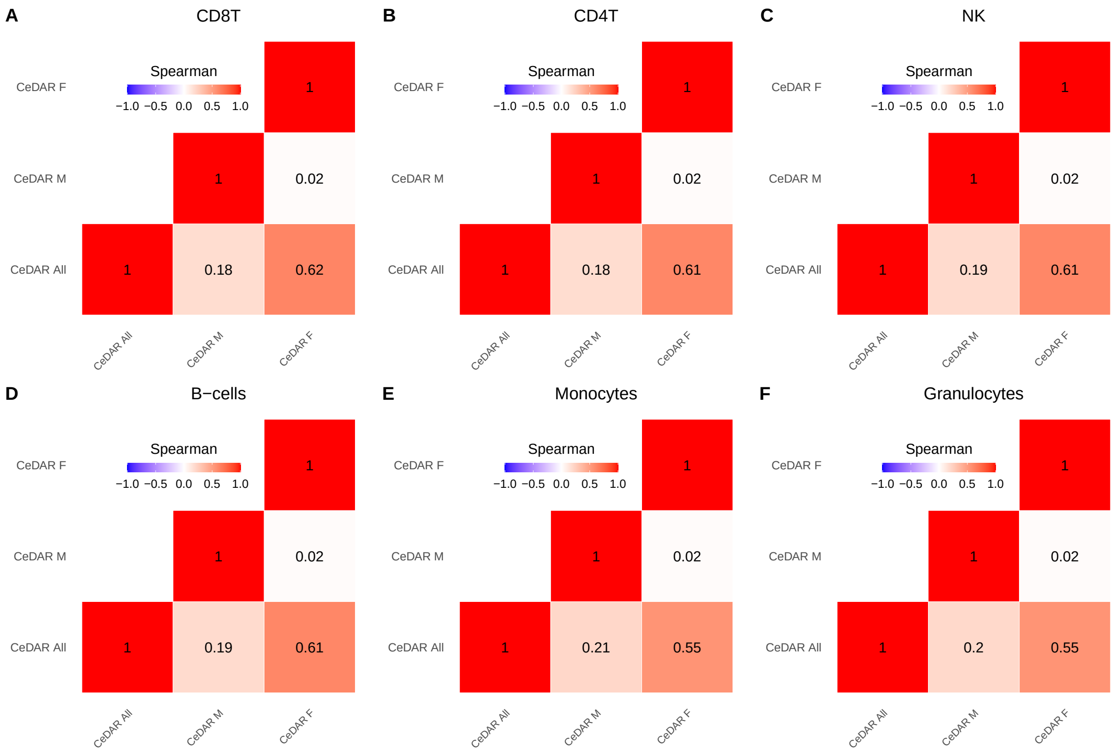

Figure S45: Correlation heatmap comparing the three subsets in CeDAR within each cell type in MDD case study. Spearman rank correlation coefficients are provided in each cell.

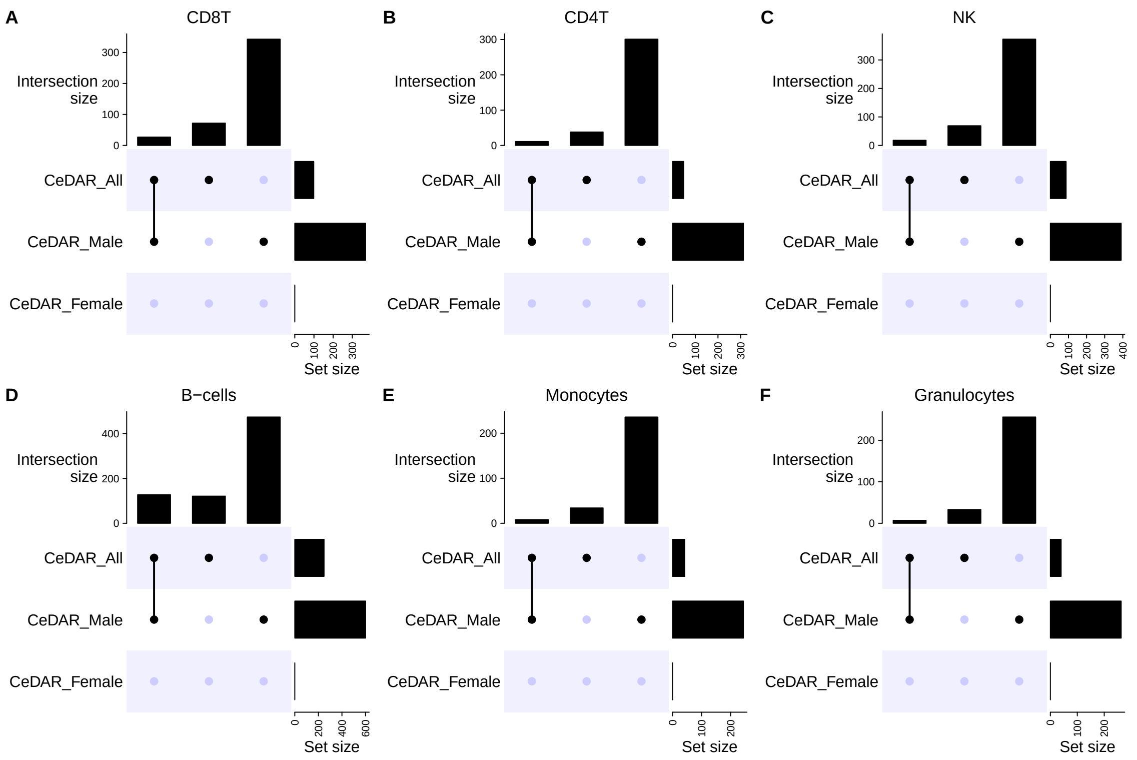

Figure S46: UpSet plot comparing the overlap among the three subsets in CeDAR in MDD case study.

Supplementary Tables

	CellDMC	TCA	HIRE	TOAST	CeDAR	*avepv*	*minpv*
cg18972751	1	0	1	1	1	1	1
cg09327855	1	0	1	1	1	1	1
cg03055671	0	0	0	0	0	0	0
cg06613783	1	0	1	1	1	1	1
cg07285641	1	0	0	1	0	1	1
cg01619562	0	0	0	0	0	0	0
cg01810713	1	0	1	1	1	1	1
cg04033022	0	0	1	0	0	0	0
cg00253346	1	0	1	1	1	1	1
cg08271031	0	0	0	0	0	0	0
Median rank	24543.5	185191	85205	24472.5	25168.25	18497.5	24457.25

Table S1: Overlap between CpGs identified in B-cells at FDR<0.01 in rheumatoid arthritis case study *versus* the list of 10 CpGs reported in Julia et al. [**?**]. Last row reports the median rank of these 10 CpGs by each method.

	CD8T	CD4T	NK	B-cells	Mono	Gran
CellDMC	5	17	3	3	0	0
TCA	1	13	0	0	0	1
HIRE	0	0	3	0	11	1
TOAST	6	17	3	3	0	0
CeDAR	4	15	3	5	0	1
avepv	4	16	0	7	0	0
minpv	3	15	0	6	0	0

Table S2: Number of KEGG gene sets identified at FDR<0.01 in rheumatoid arthritis case study.

	CD8T	CD4T	NK	B-cells	Mono	Gran
CellDMC	8	394	26	241	0	0
TCA	2	240	2	24	0	0
HIRE	0	124	155	68	265	70
TOAST	7	405	27	245	0	0
CeDAR	24	419	41	465	3	0
avepv	7	329	13	399	0	1
minpv	9	387	2	400	0	0

Table S3: Number of GO gene sets identified at FDR<0.01 in rheumatoid arthritis case study.

Tables S4 and S5 are included as separate excel files.

Table S4 Legend: List of significant KEGG gene sets identified by each method across different cell types in rheumatoid arthritis case study.

Table S5 Legend: List of significant GO gene sets identified by each method across different cell types in rheumatoid arthritis case study.

All	CD8T	CD4T	NK	B-cells	Mono	Gran
CellDMC	2	0	1	2	0	0
TCA	2	0	0	2	0	0
HIRE	0	4	0	0	4	0
TOAST	2	0	0	3	2	0
CeDAR	1	0	0	1	0	2
avepv	2	0	0	3	0	0
minpv	1	0	1	2	0	0
Male	CD8T	CD4T	NK	B-cells	Mono	Gran
CellDMC	0	0	0	1	1	0
TCA	0	0	0	0	0	0
HIRE	0	0	0	5	0	1
TOAST	3	0	0	0	0	1
CeDAR	0	0	0	1	0	0
avepv	0	0	0	0	0	0
minpv	0	0	0	0	0	0
Female	CD8T	CD4T	NK	B-cells	Mono	Gran
CellDMC	0	0	0	1	0	0
TCA	3	0	0	3	0	0
HIRE	1	0	0	0	0	0
TOAST	2	2	1	4	0	0
CeDAR	6	3	0	0	4	2
avepv	7	3	0	3	0	0
minpv	3	0	0	2	0	0

Table S6: Number of KEGG gene sets identified at FDR<0.1 in MDD case study.

All	CD8T	CD4T	NK	B-cells	Mono	Gran
CellDMC	0	0	4	1	4	3
TCA	18	0	0	0	0	0
HIRE	0	0	8	1	86	14
TOAST	7	0	0	0	2	0
CeDAR	1	0	0	0	3	0
avepv	21	0	0	0	0	0
minpv	7	0	0	0	0	0
Male	CD8T	CD4T	NK	B-cells	Mono	Gran
CellDMC	2	0	4	0	4	0
TCA	0	0	0	0	0	1
HIRE	0	0	0	32	0	0
TOAST	0	1	0	0	0	2
CeDAR	0	0	9	0	0	0
avepv	2	0	0	0	0	2
minpv	1	0	0	1	4	0
Female	CD8T	CD4T	NK	B-cells	Mono	Gran
CellDMC	0	0	0	2	0	4
TCA	3	1	0	0	6	7
HIRE	0	0	0	0	0	3
TOAST	3	1	0	0	18	6
CeDAR	0	0	0	0	1	0
avepv	0	2	0	0	7	6
minpv	0	1	0	1	4	7

Table S7: Number of GO gene sets identified at FDR<0.1 in MDD case study.
